# Separability of the Coupled-Cluster Excited State Equations: The Case of Excitonic Couplings

**DOI:** 10.1021/acs.jpca.6c00608

**Published:** 2026-06-04

**Authors:** Andreas Köhn

**Affiliations:** Institute for Theoretical Chemistry, 9149 University of Stuttgart, Paffenwaldring 55, D-70569 Stuttgart, Germany

## Abstract

Coupled-cluster theory provides an accurate description of electronic ground and excited states. While it has been rigorously established that the coupled-cluster ground state energy is size extensive and local excitations are size intensive in the thermodynamic limit, the separability of other properties in coupled-cluster theory is subject to known limitations. In particular, it was shown [Stanton, *J. Chem. Phys.* **1994,** *101*, 8928–8937] that multicenter two-particle density matrices are not asymptotically separable in general. In the present work, an analogous analysis is applied to the excitonic coupling of local excitations of two separate molecules, using both the equation-of-motion (EOM) and linear-response (LR) formalism. It is shown that for both formalisms the two-electron transition density associated with the excitonic coupling does not exactly separate into a product of local one-electron transition densities. Numerical examples are provided for Ne_2_, (CO)_2_, and (C_2_H_4_)_2_, using coupled-cluster expansions up to single, double, triple, and quadruple excitations (CCSDTQ). Small but noticeable deviations on the order of 5–10% are reported for approximations like CCSD and its second-order approximate variant CC2. As soon as triple excitations are included, the separability error becomes significantly smaller. Exploratory computations for the coupling of larger chromophores such as perylene or cumarine dyes using the CC2 method indicate that the separability error can grow up to orders of 20%. While these deviations are typically below the error margin of this method, the separability error could be of relevance in the design and benchmarking of local coupled-cluster approaches for excited states.

## Graphical abstract

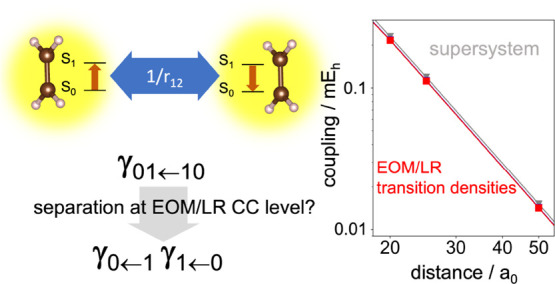

## 1 Introduction

Coupled-cluster (CC) theory^1–3^ provides a versatile framework for accurate computations of molecular electronic wave functions. One particular property of CC theory is the correct separability of the total energy, which ensures a consistent accuracy irrespective of the size of the system.^4–6^ Electronic excited states can be addressed in different ways within CC theory:^7–9^ One formulation is known as equation-of-motion (EOM) approach,^10–13^ which employs a configuration-interaction-like representation of the wave functions, based on a similarity-transformed Hamiltonian.^14^ The linear-response (LR) approach,^15–18^ on the other hand, is based on the analysis of the time-dependent response of the ground-state CC wave function and identifies excited states and their properties from the poles and residuals of the CC response functions.^19^ Both approaches are equivalent for excitation energies but lead to different expressions for properties, including transition moments.^20^

Transition moments are of central interest for a quantitative theory of molecular photophysics. Here, we focus on molecular interactions in electronic excited states, particularly the phenomenon of resonance energy transfer,^21,22^ which describes the transfer of excitation energy from one molecular entity to another, according to the scheme

$$A^{*}+B\to A+B^{*}$$

In his seminal work, Förster^23,24^ worked out the quantum-mechanical origin of the phenomenon and showed its relation to the emission and absorption spectra of the donor and acceptor molecule, respectively. Energy transfer is of particular importance for light harvesting in plants^25^ and may be used for determining distances on a molecular scale.^26^ In the limit of strongly coupled dimers, the molecular interaction becomes observable as excitonic splitting, although it should be noted that direct comparison to experiment also requires taking into account molecular vibrations.^27–29^

The accurate prediction of the electronic transition matrix elements behind these processes, which will be denoted *excitonic couplings* in the following, is desirable and has also been addressed at the coupled-cluster level, both using the second-order approximation CC2 ^30^ and the CC singles and doubles (CCSD) approach,^31^ in the latter case also including solvent effects. More recently, excitonic coupling matrix elements were used for a CC-based fragment approach to molecular eximers.^32^

These applications raise the question of separability of these interactions into contributions from the individual fragment densities. Separability of the CC solutions has been established for local excitations, in the sense that the excitation energy of a fragment is independent of the noninteracting remainder of the total system.^17,18,33–35^ Koch et al. termed this behavior *size intensivity* of the (local) excitation energy.^18^ Likewise, it was shown by Koch et al.^20^ for the linear-response formulation of the theory that the transition density of a local excitation also has this size intensivity property, whereas the EOM formulation lacks this property. Later work has established that this shortcoming of the EOM formulation is rather uncritical for typical application scenarios.^36^ For EOM excited state densities, a similar analysis was carried out by Stanton, who showed that these also remain intensive for local excitations.^37^ However, these studies revealed disconnected contributions in the projection manifold, which are unproblematic for local properties but, as pointed out by Stanton,^37^ will compromise nonlocal densities, for instance in case of charge transfer excitations across the fragments. The consequences of this have only been studied sporadically, for instance for the case of charge transfer excitations.^38^

In the present study, it will be shown that the decomposition of the excitonic coupling matrix element into molecular contributions of donor and acceptor requires the separability of a nonlocal two-electron transition density. We revisit the separability properties of CC for excited states, building on earlier work of Stanton^37^ and Koch et al.^20^ on the separability properties of the EOM and LR one-electron transition properties. We will consider both the EOM and the LR framework and show that for truncated CC expansions the separability of the two-electron transition densities connected to excitonic couplings is only approximately fulfilled. Numerical evidence is provided for a selection of examples using methods of the CC hierarchy up to single, double, triple, and quadruple excitations (CCSDTQ). In addition, the consequences for the excitonic coupling of larger systems, such as perylene or cumarine dyes, are explored at the CC2 level of theory.

## 2 Theory

### 2.1 Coupled-Cluster Ground State Equations

In CC theory, the electronic wave function in the clamped nuclei approximation is expanded as

$$\left|\Psi_{0}\rangle =e^{\hat{T}}|\Phi_{0}\right\rangle$$ 1

where |Φ_0_⟩ is a reference determinant and

$$\hat{T}=\sum_{\rho }t_{\rho }{\hat{\tau }}_{\rho }$$ 2

the cluster operator. *T̂* entails amplitudes *t*_ρ_ and particle-hole excitation operators τ̂_ρ_, where the index ρ runs over the excitation manifold. Different approximations of the theory can be defined by restricting {τ̂_ρ_} to a maximum excitation rank, e.g. single and double excitations in the CCSD approximation.

A central quantity in the usual connected formulation of coupled-cluster theory is the similarity transformed (electronic clamped-nuclei) Hamiltonian *H̅* = *e* ^–*T̂*^ *Ĥe* ^*T̂*^. The coupled-cluster ground state energy can then be expressed as

$$E_{0}=\langle \Phi_{0}|\overset{-}{H}|\Phi_{0}\rangle$$ 3

and equations for determining the amplitudes are obtained by projection to exited configurations |Φ_ρ_⟩ = *τ̂*_ρ_|Φ_0_⟩:

$$0=\langle \Phi_{\rho }|\overset{-}{H}|\Phi_{0}\rangle$$ 4

For the computation of properties, it is advantageous to formulate a stationary energy functional^39^

$$L_{0}=\langle \Phi_{0}|(1+\hat{\Lambda })\overset{-}{H}|\Phi_{0}\rangle$$ 5

where the operator Λ̂ is defined such that ⟨Φ_0_|Λ̂ = ∑_ρ_λ_ρ_⟨Φ_ρ_|. The energy functional is subject to the stationary conditions $\partial_{\lambda_{\rho }}L_{0}=0$ , which recovers the amplitude equations, eq 4, and $\partial_{t_{\rho }}L_{0}=0$ , which gives a linear set of equations from which Λ̂ can be determined (Λ equations).^39^

### 2.2 Excited States: Equation-of-Motion Approach

In the equation-of-motion (EOM) formulation,^14^ all desired energies and states are obtained as eigenvalues and -functions of the similarity transformed Hamiltonian, projected to the particle-hole basis (including the reference determinant) that corresponds to the same excitation manifold that was also considered for setting up the cluster operator. Due to the non-Hermiticity of the problem, this leads to a set of right and left-hand equations:

$$\sum_{\sigma }\langle \Phi_{\rho }|(\overset{-}{H}-E_{n})|\Phi_{\sigma }\rangle R_{\sigma }^{\left(n\right)}=0$$ 6

$$\sum_{\rho }L_{\rho }^{\left(n\right)}\langle \Phi_{\rho }|(\overset{-}{H}-E_{n})|\Phi_{\sigma }\rangle =0$$ 7

The resulting eigenvectors represent a set of right and left EOM-CC wave functions

$$|\Psi_{n}\rangle =e^{\hat{T}}{\hat{R}}^{\left(n\right)}|\Phi_{0}\rangle =e^{\hat{T}}\sum_{\rho }|\Phi_{\rho }\rangle R_{\rho }^{\left(n\right)}$$ 8

$$\langle {\tilde{\Psi }}_{n}|=\langle \Phi_{0}|{\hat{L}}^{\left(n\right)}e^{-\hat{T}}=\sum_{\rho }L_{\rho }^{\left(n\right)}\langle \Phi_{\rho }|e^{-\hat{T}}$$ 9

which are normalized as

$$\langle {\tilde{\Psi }}_{m}|\Psi_{n}\rangle =\sum_{\rho }L_{\rho }^{\left(m\right)}R_{\rho }^{\left(n\right)}=\delta_{mn}$$ 10

As the lowest eigenvalue of the EOM matrix, the CC ground state energy is recovered, with corresponding right and left eigenvectors **R** ^(0)^ = (1, **0**)^*T*^ and **L** ^(0)^ = (1, **Λ**), respectively, where **Λ** is a vector consisting of the amplitudes of the Λ̂ operator. Usually the energy scale is shifted by the CC ground state energy *E*_0_, such that the solutions of the EOM problem yield excitation energies ω_*n*0_ = *E*_*n*_ – *E*_0_ as eigenvalues.

Transition moments are straightforwardly defined as transition matrix elements of the EOM states. In particular, for the transition matrix elements of an operator *X̂* involving the ground state we get

$$X_{n0}^{\mathrm{EOM}}=\langle {\tilde{\Psi }}_{n}|\hat{X}|\Psi_{0}\rangle =\sum_{\rho }L_{\rho }^{\left(n\right)}\langle \Phi_{\rho }|\overset{-}{X}|\Phi_{0}\rangle$$ 11

­(which we will call *n*0-transition moment) and

$$X_{0n}^{\mathrm{EOM}}=\langle {\tilde{\Psi }}_{0}|\hat{X}|\Psi_{n}\rangle =\sum_{\sigma }\langle \Phi_{0}|(1+\hat{\Lambda })\overset{-}{X}|\Phi_{\sigma }\rangle R_{\sigma }^{\left(n\right)}$$ 12

­(called 0*n*-transition moment in the following).^40^ In both expressions we have used the abbreviation *X̅* = *e* ^–*T̂*^ *X̂e* ^*T̂*^. The transition moments are not the adjoints of each other, therefore only the transition strength

$$\left|X_{0n}{|}^{2}=\langle {\tilde{\Psi }}_{0}|\hat{X}|\Psi_{n}\rangle \langle {\tilde{\Psi }}_{n}|\hat{X}|\Psi_{0}\right\rangle$$ 13

is uniquely defined.^14^

### 2.3 Excited States: Linear Reponse Approach

Linear response (LR) theory provides an alternative route to excited states in CC theory.^15–20,41,42^ In this case, excitation energies are identified as the poles of the linear response function, leading to the eigenvalue problem of the matrix

$$\begin{array}{cc} A_{\rho \sigma }=\langle \Phi_{\rho }|[\overset{-}{H},{\hat{\tau }}_{\sigma }]|\Phi_{0}\rangle =\langle \Phi_{\rho }|(\overset{-}{H}-E_{0})|\Phi_{\sigma }\rangle , & \rho ,\sigma \neq 0. \end{array}$$ 14

This matrix is the first derivative of the residual equation, eq 4, with respect to the cluster amplitudes, and is therefore also addressed as Jacobian. As indicated in the second equality of eq 14, the Jacobian coincides with the excited state part of the EOM matrix, shifted by the CC ground state energy *E*_0_. The eigenvalues ω_*n*0_ = *E*_*n*_ – *E*_0_ therefore correspond to the same excitation energies as predicted by EOM theory.

Transition moments can be identified from the poles of the linear response function,^17,20^ leading in case of the *n*0-transition moment to the same expression as EOM theory

$$X_{n0}^{\mathrm{LR}}=X_{n0}^{\mathrm{EOM}}$$ 15

­(see also eq 11), while for the 0*n*-transition moment a different expression is obtained:

$$X_{0n}^{\mathrm{LR}}=\sum_{\sigma >0}\langle \Phi_{0}|(1+\hat{\Lambda })[\overset{-}{X},{\hat{\tau }}_{\sigma }]|\Phi_{0}\rangle R_{\sigma }^{\left(n\right)}+\sum_{\rho >0}M_{\rho }^{\left(0n\right)}\langle \Phi_{\rho }|\overset{-}{X}|\Phi_{0}\rangle$$ 16

The first contribution in eq 16 is similar to the EOM transition moment, eq 12, but the commutator in eq 16 restricts the expression to connected terms. It thereby excludes some terms that are present in the EOM expression, which describe a response of the cluster amplitudes due to the perturbing operator (see Supporting Information). Such a contribution is instead included by the second term of the LR expression. In fact, the expression that is directly extracted from the linear response function, explicitly contains a set of perturbed cluster amplitudes.^17^ This perturbed amplitudes are then replaced by a perturbation independent set of amplitudes *M*_ρ_^(0*n*)^, obtained by solving the linear set of equations:^41^

$$\sum_{\rho >0}M_{\rho }^{\left(0n\right)}(\langle \Phi_{\rho }|[\mathrm{H̅},{\hat{\tau }}_{\sigma }]|\Phi_{0}\rangle +\omega_{n0}\delta_{\rho \sigma })=-\langle \Phi_{0}|(1+\hat{\Lambda })[[\overset{-}{H},{\hat{R}}^{\left(n\right)}],{\hat{\tau }}_{\sigma }]|\Phi_{0}\rangle$$ 17

It can be shown analytically,^20^ that both formulations (EOM and LR) converge to the same limit in case of a full cluster expansion, but for truncated expansions they lead to different results.

## 3 Excitonic Coupling and Separability

### 3.1 Exact States

We consider two separated molecules A and B with a separable Hamiltonian *Ĥ* = *Ĥ*_A_ + *Ĥ*_B_ and assume that we know the exact solutions of the subsystems, |Ψ_*n*_^A^⟩ and |Ψ_*n*_^B^⟩. The exact states of the noninteracting supersystem are separable in this case. Of particular interest are the two locally excited states with either an excitation on A while B is in the ground state, |Ψ_1_^A^Ψ_0_^B^⟩, or the opposite case |Ψ_0_^A^Ψ_1_^B^⟩. Here, the notation with two wave functions inside a ket vector implies an antisymmetric direct product.

Further, we introduce a perturbation that couples the two systems via a Coulomb interaction. We write this operator as

$${\hat{H}}_{\mathrm{AB}}=\sum_{pqrs}{\hat{\gamma }}_{pq}^{A}\langle pr∥qs\rangle {\hat{\gamma }}_{rs}^{B}$$ 18

where

⟨pr∥qs⟩=⟨pr|qs⟩−⟨ps|qr⟩ 19

is the antisymmetrised form of the Coulomb integral

$$\left\langle pr|qs\rangle =\delta_{\sigma_{p}\sigma_{q}}\delta_{\sigma_{r}\sigma_{s}}\int d^{3}r_{1}d^{3}r_{2}\phi_{p}^{*}(r_{1})\phi_{r}^{*}(r_{2})\frac{1}{r_{12}}\phi_{q}(r_{1})\phi_{s}(r_{2}\right)$$ 20

expressed in terms of spatial orbitals ϕ_*p*_(**r**) and corresponding spin quantum numbers σ_*p*_. The symbols *γ̂*_*pr*_ are the corresponding reduced one-particle density operators, represented in terms of orbital pairs.

To first-order perturbation theory, the interaction matrix element between the locally excited states is

$$V_{\mathrm{AB}}=\langle \Psi_{1}^{A}\Psi_{0}^{B}|{\hat{H}}_{\mathrm{AB}}|\Psi_{0}^{A}\Psi_{1}^{B}\rangle =\sum_{pqrs}\langle \Psi_{1}^{A}|{\hat{\gamma }}_{pq}^{A}|\Psi_{0}^{A}\rangle \langle pr∥qs\rangle \langle \Psi_{0}^{B}|{\hat{\gamma }}_{rs}^{B}|\Psi_{1}^{B}\rangle$$ 21

This matrix element is the leading-order coupling between the two transitions on molecules A and B. It is the core interaction that is required for describing energy transfer processes and excitonic splittings and will be addressed as excitonic coupling in the following. The excitonic coupling can be expressed in terms of one-particle transition densities on either fragment, coupled by the Coulomb interaction. The exchange interaction that is also present in the above expression is negligible for sufficiently separated fragments. It starts to play a role as soon as the wave functions of A and B start to overlap; in this case, however, also reorthogonalisation and charge-transfer matrix elements begin to play a role, as well.^43^ Here, we are only interested in the long-range limit.

For sufficiently large separation of the molecular centers, described by a distance vector **R**_AB_, the coupling can be approximated by the interaction of dipole transitions of the subsystems, **μ**_10_^A^ = ⟨Ψ_1_^A^| ***μ̂*** |Ψ_0_^A^⟩ and **μ**_01_^B^ = ⟨Ψ_0_^B^| ***μ̂*** |Ψ_1_^B^⟩, giving

$$V_{\mathrm{AB}}\approx \frac{\mu_{10}^{A}\cdot \mu_{01}^{B}-3(\mu_{10}^{A}\cdot n_{\mathrm{AB}})(\mu_{01}^{B}\cdot n_{\mathrm{AB}})}{R_{\mathrm{AB}}^{3}}$$ 22

with *R*_AB_ = ∥**R**_AB_∥ and **n**_AB_ = **R**_AB_/*R*_AB_. This expression can then be related to the emission and absorption spectra of the two systems, which is the basis of Förster theory.^23,24^

### 3.2 EOM Picture

In the following, we analyze the excitonic coupling of two well-separated molecules within EOM theory. In particular, we seek to understand, in how far the expression can be separated as suggested by the expression for exact states, eq 21. The reference determinant of the combined system can be written as the antisymmetrised product of the subsystem determinants: |Φ_0_⟩ = |Φ_0_^A^Φ_0_^B^⟩. As shown in literature,^6^ the CC equations separate and so does the ground state energy: *E*_0_ = *E*_0_^A^ + *E*_0_^B^. The cluster operator only consists of local excitations on either system A or B, *T̂* = *T̂*_A_ + *T̂*_B_ (in obvious notation) and as a consequence, the similarity transformed Hamiltonian is fully separable:

$$\overset{-}{H}={\overset{-}{H}}_{A}+{\overset{-}{H}}_{B}=e^{-{\hat{T}}_{A}}{\hat{H}}_{A}e^{{\hat{T}}_{A}}+e^{-{\hat{T}}_{B}}{\hat{H}}_{B}e^{{\hat{T}}_{B}}$$ 23

The discussion of the matrix elements of the EOM matrix, however, is a bit more intricate.^37^ We have to distinguish three types of configurations:

configurations in which only molecule A is excited, while B resides in the reference determinant, denoted by
$$|\Phi_{\rho_{A}}\rangle =|\Phi_{\rho_{A}}^{A}\Phi_{0}^{B}\rangle ;$$
the reverse case with excitations only on molecule B, denoted by
$$|\Phi_{\rho_{B}}\rangle =|\Phi_{0}^{A}\Phi_{\rho_{B}}^{B}\rangle ;$$
and configurations, which involve excitations on both molecules, denoted by
$$|\Phi_{\rho_{X}}\rangle =\left|\Phi_{\rho_{X,A}}^{A}\Phi_{\rho_{X,B}}^{B}\right\rangle$$

Details on the notation used in the following are summarized in Table 1. For the last case, we have to further distinguish excitations that keep the charge conserved on each subsystem and those with a transfer of charge; the latter, however, are fully decoupled and not of interest here, see also ref 37. Note also that for the case of truncated expansions (the only case relevant in practice), the excitation manifolds are restricted to a maximum total excitation, such that, for instance, the manifold enumerated by ρ_X,A_ is only a subset of the excitations on fragment A (enumerated by ρ_A_).

**Table 1:** Notation Used in This Work for the Different Types of Configurations and Corresponding Excitation Vectors^a^

type	description	configurations	vectors	vector elements
A	exc. on A only	$|\Phi_{\rho_{A}}\rangle =\left|\Phi_{\rho_{A}}^{A}\Phi_{0}^{B}\right\rangle$	**R**_A_	*R*_ρ_A__, *R*_σ_A__
B	exc. on B only	$|\Phi_{\rho_{B}}\rangle =\left|\Phi_{0}^{A}\Phi_{\rho_{B}}^{B}\right\rangle$	**R**_B_	*R*_ρ_B__, *R*_σ_B__
X	exc. on both A and B	$|\Phi_{\rho_{X}}\rangle =\left|\Phi_{\rho_{X,A}}^{A}\Phi_{\rho_{X,B}}^{B}\right\rangle$	**R**_X_	*R*_ρ_X__, *R*_σ_X__

a For the mixed case, also the indices *ρ*_X,A_ and *ρ*_X,B_ are used to enumerate the partial contributions to mixed excitations that reside on the subsystem A or B, respectively.

Using the locality of the similarity transformed Hamiltonian and the orthogonality of configurations, the EOM matrix reduces to the following block matrix representation (see ref 37. and Supporting Information):

$$\begin{array}{rl} \overset{-}{H}=\left(\begin{array}{llll} {\overset{-}{H}}_{00} & {\overset{-}{H}}_{0A} & {\overset{-}{H}}_{0B} & 0\\ 0 & {\overset{-}{H}}_{\mathrm{AA}} & 0 & {\overset{-}{H}}_{\mathrm{AX}}\\ 0 & 0 & {\overset{-}{H}}_{\mathrm{BB}} & {\overset{-}{H}}_{\mathrm{BX}}\\ 0 & 0 & 0 & {\overset{-}{H}}_{\mathrm{XX}} \end{array}\right) & \end{array}$$ 24

Due to the triangular block matrix form, the right-hand side equations separate and the excitation vectors are of the form (see Supporting Information)­

$$R^{(A),T}=(R_{0}^{\left(A\right)},R_{A}^{\left(A\right)},0,0{)}^{T}$$ 25

$$R^{(B),T}=(R_{0}^{\left(B\right)},0,R_{B}^{\left(B\right)},0{)}^{T}$$ 26

i.e. they represent local excitations on either fragment A or B. Note that the superscripts like (A) indicate the excitation, while the subscripts indicate the involved configuration, see also Table 1. The contributions *R*_0_^(A)^ and *R*_0_^(B)^ that involve the reference determinant are only relevant for excited states of the same spatial and spin symmetry as the ground state.

The left-hand eigenvalue problem, however, is not fully separable (see Supporting Information). The excitation vectors have contributions from the delocalized X space:

$$L^{\left(A\right)}=(0,L_{A}^{\left(A\right)},0,L_{X}^{\left(A\right)})$$ 27

$$L^{\left(B\right)}=(0,0,L_{B}^{\left(B\right)},L_{X}^{\left(B\right)})$$ 28

where the equations can be recast such that, for instance, **L**_A_^(A)^ is obtained as the left eigenvector of **H̅**_A_, and the X contribution is determined by this linear set of equations:

$$L_{X}^{\left(A\right)}(H_{\mathrm{XX}}-E_{A}1_{X})=-L_{A}^{\left(A\right)}{\overset{-}{H}}_{\mathrm{AX}}$$ 29

As discussed in ref 37, the right-hand side of this equation factorizes into contributions that are purely localized on either fragment

$$L_{A}^{\left(A\right)}{\overset{-}{H}}_{\mathrm{AX}}=\left(\sum_{\rho_{A}}L_{\rho_{A}}^{\left(A\right)}\langle \Phi_{\rho_{A}}^{A}|\Phi_{\sigma_{X,A}}^{A}\rangle \right)\langle \Phi_{0}^{B}|{\overset{-}{H}}_{B}|\Phi_{\sigma_{X,B}}^{B}\rangle$$ 30

while the matrix-vector product factorizes as

$$\begin{array}{rl} L_{X}^{\left(A\right)}{\overset{-}{H}}_{\mathrm{XX}} & =\left(\sum_{\rho_{X,A}}L_{\rho_{X,A}}^{\left(A\right)}\langle \Phi_{\rho_{X,A}}^{A}|{\overset{-}{H}}_{A}|\Phi_{\sigma_{X,A}}^{A}\rangle \right)L_{\sigma_{X,B}}^{\left(A\right)}\\ & +L_{\sigma_{X,A}}^{\left(A\right)}\left(\sum_{\rho_{X,B}}L_{\rho_{X,B}}^{\left(X\right)}\langle \Phi_{\rho_{X,B}}^{B}|{\overset{-}{H}}_{B}|\Phi_{\sigma_{X,B}}^{B}\rangle \right) \end{array}$$ 31

which is the direct product of the left-hand matrix-vector products, restricted to the maximum excitation level. This expression will only factorize exactly in the full coupled-cluster limit (see Supporting Information). From the arguments presented so far, we can approximately describe the mixed left excitation vector as the direct product **L**_X_^(A)^ ≈ **L**_A_^(A)^ ⊗ **Λ**_B_, but truncated to the maximum excitation level of the considered approximation.^37^

Based on this analysis of the excitation vectors, we can now investigate the separability of the excitonic coupling matrix element. Adding the perturbation, eq 18, to the Hamiltonian, first-order perturbation theory gives

$$V_{\mathrm{AB}}^{\mathrm{EOM}}=L^{\left(A\right)}{\overset{-}{H}}^{\left(1\right)}R^{\left(B\right)}=L_{A}^{\left(A\right)}{\overset{-}{H}}_{\mathrm{AB}}^{\left(1\right)}R_{B}^{\left(B\right)}+L_{A}^{\left(A\right)}{\overset{-}{H}}_{A0}^{\left(1\right)}R_{0}^{\left(B\right)}+L_{X}^{\left(A\right)}{\overset{-}{H}}_{\mathrm{XB}}^{\left(1\right)}R_{B}^{\left(B\right)}+L_{X}^{\left(A\right)}{\overset{-}{H}}_{X0}^{\left(1\right)}R_{0}^{\left(B\right)}$$ 32

where again the *R*_0_^(B)^ contributions are only relevant for totally symmetric transitions. In the following, we absorb the *R*_0_^(B)^-containing terms into the **R**_*B*_^(B)^ terms by including |Φ_0_^B^⟩ into the manifold {|Φ_σ_B__^B^⟩}. More explicitly, we get the following two contributions: First, the coupling between the left-hand excitation on fragment A and the right-hand excitation on fragment B:

$$L_{A}^{\left(A\right)}{\overset{-}{H}}_{\mathrm{AB}}R_{B}^{\left(B\right)}=\sum_{\rho_{A},\sigma_{B}}L_{\rho_{A}}^{\left(A\right)}\langle \Phi_{\rho_{A}}^{A}\Phi_{0}^{B}|{\overset{-}{H}}_{\mathrm{AB}}|\Phi_{0}^{A}\Phi_{\sigma_{B}}^{B}\rangle R_{\sigma_{B}}^{\left(B\right)}$$ 33

$$=\sum_{pqrs}\left(\sum_{\rho_{A}}L_{\rho_{A}}^{\left(A\right)}\langle \Phi_{\rho_{A}}^{A}|{\overset{-}{\gamma }}_{pr}^{A}|\Phi_{0}^{A}\rangle \right)\langle pr∥qs\rangle \left(\sum_{\sigma_{B}}\langle \Phi_{0}^{B}|{\overset{®}{\gamma }}_{qs}^{B}|\Phi_{\sigma_{B}}^{B}\rangle R_{\sigma_{B}}^{\left(B\right)}\right)$$ 34

Here, we have used the abbreviations γ̅_*pq*_^A^ = *e* ^–*T̂*_A_^ *γ̂*_*pq*_^A^ *e* ^*T̂*_A_^ and γ̅_*pq*_^B^ = *e* ^–*T̂*_B_^ *γ̂*_*pq*_^B^ *e* ^*T̂*_B_^. This expression is in line with the expectation from exact theory, the local contributions from either fragments are coupled via the Coulomb interaction. However, only the *n*0 transition moment is recovered, compare the contents of the first parentheses of eq 34 to eq 11, while the 0*n* transition moment is incomplete (second parentheses in comparison to eq 12, which misses the Λ̂ contribution). These contributions therefore have to be identified in the second contribution to *V*_AB_^EOM^. To proceed, we use that **L**_X_^(A)^ is disconnected and write these amplitudes in terms of a product of local contributions *L̃*_ρ_X,A__^(A)^ and *L̃*_ρ_X,B__^(A)^:

$$L_{X}^{\left(A\right)}{\overset{-}{H}}_{\mathrm{XB}}R_{B}^{\left(B\right)}=\sum_{\rho_{X,A},\rho_{X,B},\sigma_{B}}{\tilde{L}}_{\rho_{X,A}}^{\left(A\right)}{\tilde{L}}_{\rho_{X,B}}^{\left(A\right)}\langle \Phi_{\rho_{X,A}}^{A}\Phi_{\rho_{X,B}}^{B}|{\overset{-}{H}}_{\mathrm{AB}}|\Phi_{0}^{A}\Phi_{\sigma_{B}}^{B}\rangle R_{\sigma_{B}}^{\left(B\right)}$$ 35

=∑pqrs(∑ρX,AL~ρX,A(A)⟨ΦρAA|γ−pqA|Φ0A⟩)⟨pr∥qs⟩×(∑ρX,BσBL~ρX,B(A)⟨ΦρX,BB|γ−rsB|ΦσBB⟩RσB(B)) 36

The sum of the two contributions, eqs 34 and 36, may be compared to the expression obtained by coupling the EOM transition densities of fragments A and B:

V~ABEOM=∑pqrs(∑ρALρA(A)⟨ΦρAA|γ−pqA|Φ0A⟩)⟨pr∥qs⟩×(∑σB⟨Φ0B|(1+Λ^B)γ−rsB|ΦσBB⟩RσB(B)) 37

Here, summation over σ_B_ also includes the reference configuration, if required. The only discrepancy to the supersystem expression comes from the incomplete representation of **L** ^(A)^ ⊗ **Λ**_*B*_ by **L**_X_^(*A*)^. For example, for CCSD, **L**_X_^(A)^ is truncated to single and double excitations and will thus only be able to represent a product of single excitations of **L** ^(A)^ ⊗ **Λ**_*B*_. For the coupling of single-excitation dominated electronic transitions, this may already recover the main contributions; higher-level approximations will further improve this term. Nevertheless, it becomes clear that a full numerical match requires a full CC expansion.

### 3.3 Response Theory Picture

Under the LR perspective, basically the same problems occur. It is nevertheless worthwhile, to highlight a few details. As discussed above, the in LR theory the eigenvalues and eigenvectors of the Jacobian are central for determining the excitation energies, where the Jacobian equals the shifted EOM matrix, but without the matrix elements that describe a coupling to the ground state. For a supersystem of two noninteracting systems, we can write

$$\begin{array}{rl} A=\left(\begin{array}{lll} A_{\mathrm{AA}} & 0 & A_{\mathrm{AX}}\\ 0 & A_{\mathrm{BB}} & A_{\mathrm{BX}}\\ 0 & 0 & A_{\mathrm{XX}} \end{array}\right) & \end{array}$$ 38

with obvious analogy to the EOM matrix, eq 24. This matrix was also investigated in ref 20 to prove the intensivity of the LR transition moments of noninteracting molecules. The findings are analogous to the EOM case with the same solutions, but without the *R*_0_ contributions. For the right-hand excitation vectors, the result is fully separable

$$R^{(A),T}=(R_{A}^{\left(A\right)},0,0{)}^{T}$$ 39

$$R^{(B),T}=(0,R_{B}^{\left(B\right)},0{)}^{T}$$ 40

while for the left-hand excitation vectors the same disconnected contributions are found, which appear as delocalized excitations in the X space:

$$L^{\left(A\right)}=(L_{A}^{\left(A\right)},0,L_{X}^{\left(A\right)})$$ 41

$$L^{\left(B\right)}=(0,L_{B}^{\left(B\right)},L_{X}^{\left(B\right)})$$ 42

These extra contributions are of no harm concerning the separability of the local excitations and transition moments,^17,20^ but their role in the excitonic coupling matrix elements has to be analyzed, in analogy to the EOM case, Section 3.2.

Unlike the EOM formulation, the ground state is not explicitly present in the Jacobian, but has to be considered as well. We can understand the excitonic coupling as a two-electron transition moment between the two locally excited states, with the Coulombic coupling, eq 18, as perturbation. Such an expression can be formally extracted from the poles of the quadratic response function,^42^ and reads

$$V_{\mathrm{AB}}^{\mathrm{LR}}=L^{\left(A\right)}A_{\mathrm{AB}}^{\left(1\right)}(\omega_{\mathrm{AB}})R^{\left(B\right)}$$ 43

with

$$A_{\mathrm{AB}}^{\left(1\right)}(\omega_{\mathrm{AB}}):\langle \Phi_{\rho }|[{\overset{-}{H}}_{\mathrm{AB}},{\hat{\tau }}_{\rho }]|\Phi_{0}\rangle +\langle \Phi_{\rho }|[[{\overset{-}{H}}_{A}+{\overset{-}{H}}_{A},{\hat{T}}^{\left(1\right)}(\omega_{\mathrm{AB}})],{\hat{\tau }}_{\rho }]|\Phi_{0}\rangle$$ 44

where ω_AB_ = ω_A0_ – ω_B0_ = *E*_A_ – *E*_B_ is the difference in transition energies. (Note that in the numerical part of this work we will focus on examples with equal excitation energies such that ω_AB_ vanishes.) We see that this expression not only comprises the perturbing operator, but also the perturbed amplitudes of the ground-state. These amplitudes, which explicitly depend on the perturbing operator, can be replaced by a set of amplitudes that may be interpreted as Lagrange multipliers for the condition that the ground state equations remain fulfilled under all perturbations.^41,42^ This allows rewriting the transition moment expression as

$$V_{\mathrm{AB}}^{\mathrm{LR}}=\sum_{\rho \sigma }L_{\rho }^{\left(A\right)}\langle \Phi_{\rho }|[{\overset{-}{H}}_{\mathrm{AB}},{\hat{\tau }}_{\sigma }]|\Phi_{0}\rangle R_{\sigma }^{\left(B\right)}+\sum_{\rho }N_{\rho }^{\left(\mathrm{AB}\right)}\langle \Phi_{\rho }|{\overset{-}{H}}_{\mathrm{AB}}|\Phi_{0}\rangle$$ 45

where the amplitudes *N*_ρ_^(AB)^ are obtained as solutions of the linear set of equations

$$\sum_{\rho }N_{\rho }^{\left(\mathrm{AB}\right)}\left(\langle \Phi_{\rho }|[\overset{-}{H},{\hat{\tau }}_{\sigma }]|\Phi_{0}\rangle +\omega_{\mathrm{AB}}\delta_{\rho \sigma }\right)=-\sum_{\rho \nu }L_{\rho }^{\left(A\right)}\langle \Phi_{\rho }|[[\overset{-}{H},{\hat{\tau }}_{\nu }],{\hat{\tau }}_{\sigma }]|\Phi_{0}\rangle R_{\nu }^{\left(B\right)}$$ 46

which only depends on the zeroth-order (noninteracting) Hamiltonian. In short-hand form, we write this equation as

$$N^{\left(\mathrm{AB}\right)}(A+\omega_{\mathrm{AB}}1)=-n$$ 47

This equation can be analyzed in analogy to the equations that determine the disconnected contributions **L**_X_^(A)^, see Section 3.2. The right-hand side becomes

$$\begin{array}{rl} n_{\sigma }= & \sum_{\rho_{A}\nu_{B}}L_{\rho_{A}}^{\left(A\right)}\langle \Phi_{\rho_{A}}^{A}\Phi_{0}^{B}|[[{\overset{-}{H}}_{B},{\hat{\tau }}_{\nu_{B}}],{\hat{\tau }}_{\sigma }]|\Phi_{0}\rangle R_{\nu_{B}}^{\left(B\right)}\\ & +\sum_{\rho_{X}\nu_{B}}L_{\rho_{X}}^{\left(A\right)}\langle \Phi_{\rho_{X,A}}^{A}\Phi_{\rho_{X,B}}^{B}|[[{\overset{-}{H}}_{B},{\hat{\tau }}_{\nu_{B}}],{\hat{\tau }}_{\sigma }]|\Phi_{0}\rangle R_{\nu_{B}}^{\left(B\right)} \end{array}$$ 48

where the commutator with *τ̂*_ν_B__ effects that only terms with *H̅*_B_ survive. As a consequence, if we partition the operator *τ̂*_σ_, the contributions from *τ̂*_σ_A__ vanish as [*H̅*_B_, *τ̂*_σ_A__] = 0 and so do the contributions from *τ̂*_σ_B__, as then the resulting operator product purely operates in space B and ⟨Φ_ρ_^A^|Φ_0_^A^⟩ = 0. Therefore, the right-hand side has only nonvanishing contributions for the X space. Using the factorization of **L**_X_^(A)^ into its disconnected contributions, we can write the right-hand side vector as

$$n_{\sigma_{X}}=\left(\sum_{\rho_{A}}L_{\rho_{A}}^{\left(A\right)}\langle \Phi_{\rho_{A}}^{A}|{\hat{\tau }}_{\sigma_{X,A}}|\Phi_{0}^{A}\rangle \right)\left(\sum_{\nu_{B}}\langle \Phi_{0}^{B}|[[{\overset{-}{H}}_{B},{\hat{\tau }}_{\nu_{B}}],{\hat{\tau }}_{\sigma_{X,B}}]|\Phi_{0}^{B}\rangle R_{\nu_{B}}^{\left(B\right)}\right)+\left(\sum_{\rho_{X,A}}{\tilde{L}}_{\rho_{X,A}}^{\left(A\right)}\langle \Phi_{\rho_{X,A}}^{A}|{\hat{\tau }}_{\sigma_{X,A}}|\Phi_{0}^{A}\rangle \right)\times \left(\sum_{\rho_{X,B},\nu_{B}}{\tilde{L}}_{\rho_{X,B}}^{\left(A\right)}\langle \Phi_{\rho_{X,B}}^{B}|[[{\overset{-}{H}}_{B},{\hat{\tau }}_{\nu_{B}}],{\hat{\tau }}_{\sigma_{X,B}}]|\Phi_{0}^{\left(B\right)}\rangle R_{\nu_{B}}^{\left(B\right)}\right)$$ 49

Both terms factorize into contributions localized on either fragment A or B. Their structure suggests that, in the limit of a full cluster expansion, they can be expressed as

$$n_{\rho_{A}⊗\sigma_{B}}=L_{\rho_{A}}^{\left(A\right)}\langle \Phi_{0}^{B}|(1+{\hat{\Lambda }}_{B})[[{\overset{-}{H}}_{B},{\hat{R}}_{B}^{\left(B\right)}],{\hat{\tau }}_{\sigma_{B}}]|\Phi_{0}^{B}\rangle$$ 50

which is the **L**_A_^(A)^ excitation vector multiplied with an expression that can be identified as the right-hand side of a linear set of equations to determine the **M** vector of a 0*n* transition moment, see eq 17.

Equation 47 can be written as

$$N_{A}^{\left(\mathrm{AB}\right)}(A_{\mathrm{AA}}+\omega_{\mathrm{AB}}1)=0$$ 51

$$N_{B}^{\left(\mathrm{AB}\right)}(A_{\mathrm{BB}}+\omega_{\mathrm{AB}}1)=0$$ 52

$$N_{A}^{\left(\mathrm{AB}\right)}A_{\mathrm{AX}}+N_{B}^{\left(\mathrm{AB}\right)}A_{\mathrm{BX}}+N_{X}^{\left(\mathrm{AB}\right)}(A_{\mathrm{XX}}+\omega_{\mathrm{AB}}1)=-n_{X}$$ 53

The first two lines have only the trivial solution **N**_A_^(AB)^ = **N**_B_^(AB)^ = **0**, and the first two terms on the left-hand side of eq 53 can be eliminated. As discussed above, **n**_X_ factorizes, and **N**_X_^(AB)^ **A**_XX_ factorizes, too, in analogy to eq 31. Consequently **N**_X_^(AB)^ is fully disconnected and can be written as a product of local contributions on fragments A and B, which in the full coupled-cluster limit become **N**_X_^(AB)^ ≈ **L** ^(A)^ ⊗ **M** ^(B)^. This suggests that eq 45 can be approximately turned into an expression computed from the transition moments of the individual fragments

$${\tilde{V}}_{\mathrm{AB}}^{\mathrm{LR}}=\sum_{pqrs}\left(\sum_{\rho_{A}}L_{\rho_{A}}^{\left(A\right)}\langle \Phi_{\rho_{A}}^{A}|{\overset{-}{\gamma }}_{pq}^{A}|\Phi_{0}^{A}\rangle \right)\langle pr∥qs\rangle \times (\sum_{\sigma_{B}}\langle \Phi_{0}^{B}|(1+{\hat{\Lambda }}_{B})[{\overset{-}{\gamma }}_{rs}^{B},{\hat{\tau }}_{\sigma_{B}}]|\Phi_{0}^{B}\rangle R_{\sigma_{B}}^{\left(B\right)}+\sum_{\sigma_{B}}M_{\sigma_{B}}^{\left(0B\right)}\langle \Phi_{\sigma_{B}}|{\overset{-}{\gamma }}_{rs}^{B}|\Phi_{0}^{\left(B\right)}\rangle )$$ 54

This expression differs from its EOM analogue, eq 37, but should converge to the same limit for sufficiently large cluster expansions.

## 4 Numerical Results

### 4.1 Computational Setup

The equations for computing coupled-cluster excitation energies and transition moments in either the EOM or LR formalism were implemented with the help of the symbolic algebra approach of the GeCCo (general contraction code) program.^44^ In particular, this allows to perform computations with high-order cluster expansions. For this work, we use the coupled-cluster hierarchy reaching from CCS to CCSDTQ, including the important perturbative approximations CC2 ^45^ and CC3.^46^ Molecular integrals and Hartree–Fock orbitals were imported from Molpro.^47–49^ Some additional tests for larger systems were performed at the CC2 level using the Turbomole program package.^50–53^ In these cases, density fitting (also known as resolution of the identity trick)^54^ was applied for the evaluation of two-electron repulsion integrals.

Excitonic couplings of a sample test set of symmetric homodimers were computed (see below for details on the considered systems). In this case, assuming sufficiently large separation of the fragments and no interference with other electronic transitions, the energy splitting is directly related to the coupling matrix element as Δ*E*_AB_ = 2|*V*_AB_|. The couplings were either obtained by computing the difference between the lowest two states of the supersystem at varying distances or by coupling the transition densities (from either the EOM or LR formalism) via the Coulomb operator (including exchange terms). To this end, the (one-electron) transition densities computed in GeCCo were backtransformed into the atomic orbital basis and written to a file. If required, the densities were transformed to C_1_ symmetry and then expanded into the product space basis of the dimer. These matrices were read in by Molpro using its matrop (matrix operations) module, which can compute Coulomb (and exchange) matrices of arbitrary supplied density matrices and finally compute the trace with another density matrix (see Supporting Information for an example input). In this way, eqs 37 and 54 could be evaluated for arbitrary distances. In addition, the transition dipole matrix elements (length gauge) were used to evaluate the excitonic coupling in the dipole approximation, eq 22. As all considered cases are symmetric, coupling matrix elements were also symmetric under exchange of A and B (which they are not in general).

The following three systems were investigated at various levels of the CC hierachy:

Ne_2_: Two neon atoms are displaced along the *z* axis and the coupling of the *x* component of the P ← S transition is considered.
(CO)_2_: Each CO molecule is kept at an equilibrium distance of 2.13161*a*_0_ and the dimer is arranged collinearly along the *z* axis with the oxygen atoms oriented toward each other. This arrangement was chosen to avoid an additional energy splitting due to static dipole interactions. The reported distances were computed relative to the center of charge of the nuclei (which was an arbitrary choice; it is only relevant for the dipole approximation). For the coupling, the *x* component of the ^1^Π_u_ ← ^1^Σ_g_ transition was considered.
(C_2_H_4_)_2_: Each molecule is D_2h_ symmetric, with *r*(C=C) = 2.5512*a*_0_, *r*(C–H) = 2.0598*a*_0_ and α­(HCH) = 117.205°. The molecules are placed in a ‘stacked’ arrangement with the π-systems directed toward each other. The considered excitation is the lowest π^∗^ ← π transition.

In all cases, the aug-cc-pV*X*Z basis set (mostly with *X* = D, for Neon also with *X* = T,Q)^55,56^ set was used, along with the frozen-core approximation applied to the 1s cores.

Further tests, restricted to the CC2 method, were carried out for symmetric dimers (’stacked’ arrangement) of butadiene, cumarine 120 (7-amino-4-methyl-cumarine) and perylenediimide. These computations employed the def2-TZVPP basis^57^ sets along with the appropriate auxiliary basis sets for the density fitting.^58^ The frozen-core approximation for the 1s cores was applied, as before.

### 4.2 Excitation Energies and Transition Moments

Before discussing the excitonic splittings, we quickly review the results for the isolated molecules. Of particular interest are the differences in the transition moments computed in the EOM and LR formalism. While there is earlier work, comparing these approaches at the CCSD level,^20,36^ or investigating the convergence along the coupled-cluster hierarchy for the LR approach,^59^ a direct comparison of the convergence behavior of both approaches has not been reported so far.

In Table 2, we have collected the vertical excitation energies and transition moments, as well as the resulting oscillator strength in length gauge $f_{0n}^{l}=\frac{1}{3}\omega_{0n}|\langle 0|\hat{\mu }|n\rangle {|}^{2}$ . Concerning the transition moments, we note that their phases and normalization factors are not uniquely defined. We here report all moments as positive real quantities and using a unit normalization of the **R** vector in the spin–orbital basis, in addition to the biorthogonal norm (which only fixes the relative norm of **R** and **L**). Some programs use a different normalization (for closed-shell cases), for which the 0*n*-transition moment is larger by $\sqrt{2}$ while the *n*0-transition moment is scaled by $1/\sqrt{2}$ . As obvious from Table 2, the current choice appears more natural, as both transition moments have comparable values in this case.

**Table 2:** Vertical Excitation Energies, Transition Moments, and Resulting Oscillator Strengths of the Lowest Singlet Electronic Transitions of Ne, CO, and C_2_H_4_ ^a^

			⟨1|μ̂|0⟩/*ea*_0_	⟨0|μ̂|1⟩/*ea*_0_			osc. str.^b^	
system	method	Δ*E*/eV	EOM/LR	EOM	LR	*M* contr.^c^	EOM	LR
Ne	CCS	20.784	0.4763	0.4763	0.4717	–0.0046	0.3466	0.3433
^1^P← ^1^S	CC2	18.745	0.5163	0.5018	0.4995	–0.0051	0.3569	0.3553
	CCSD	19.041	0.5072	0.4889	0.4882	–0.0044	0.3470	0.3465
	CC3	19.240	0.5042	0.4893	0.4892	–0.0045	0.3488	0.3488
	CCSDT	19.206	0.5042	0.4891	0.4890	–0.0045	0.3481	0.3480
	CCSDTQ	19.244	0.5041	0.4887	0.4887	–0.0044	0.3485	0.3484
								
CO	CCS	9.078	0.7185	0.7185	0.6018	–0.1167	0.2296	0.1923
^1^Π_u_ ← ^1^Σ_g_	CC2	8.775	0.6598	0.6489	0.6182	–0.0975	0.1841	0.1754
	CCSD	8.721	0.6512	0.6313	0.6208	–0.0954	0.1757	0.1727
	CC3	8.622	0.6714	0.6454	0.6416	–0.0950	0.1831	0.1820
	CCSDT	8.623	0.6644	0.6378	0.6355	–0.0943	0.1790	0.1784
	CCSDTQ	8.612	0.6677	0.6371	0.6368	–0.0947	0.1795	0.1794
								
C_2_H_4_	CCS	7.056	0.7333	0.7333	0.6998	–0.0335	0.0930	0.0887
^1^B_1*u*_ ← ^1^A_g_	CC2	7.099	0.6956	0.6696	0.6712	–0.0180	0.0810	0.0812
	CCSD	7.251	0.6838	0.6554	0.6511	–0.0175	0.0796	0.0791
	CC3	7.210	0.6931	0.6510	0.6509	–0.0162	0.0797	0.0797
	CCSDT	7.220	0.6922	0.6507	0.6499	–0.0163	0.0797	0.0796
	CCSDTQ	7.228	0.6925	0.6484	0.6483	–0.0161	0.0795	0.0795

a The aug-cc-pVDZ basis was used throughout.

b Oscillator strength (length gauge); for Neon a factor 3 and for CO a factor 2 are included to account for the degeneracy of the transition.

c Lagrange multiplier contribution to the 0*n* transition moment.

The *n*0-transition moment, which involves the **L** excitation vector, is identical for EOM and LR and converges, for the selected examples, relatively quickly toward the most accurate CCSDTQ value. Only the lowest-order approximations, CCS and CC2, show more significant deviations from this limit.

For the 0*n* transition moments, different expressions are used in EOM and LR. Consequently, there are deviations up to 10% for CCS, which diminish to at most 1% for CCSD and to same value within 0.01% at the CCSDTQ level. The contribution from the **M** amplitudes, compare eq 16, remains significant all along the coupled-cluster hierarchy, in particular for CO, where it contributes nearly 15%. As argued in Section 2.3, this contribution represents the response of the ground-state cluster amplitudes. It is interesting to note that the (partially) disconnected contributions in the EOM approach lead to nearly the same values at the CCSD level and all more accurate approximations.

The CCS approximation is a special case. Here, the EOM approach is fully equivalent to the (Hermitian) configuration interaction singles approach, CIS.^60^ In this case, the 0*n* and *n*0 transition moments are the same (see Table 2). Interestingly, this symmetry is broken in the LR formulation of CCS. For the considered cases, this brings the oscillator strength a bit closer to the CCSDSQ values (except for Ne where this difference is small), but clearly the number of test cases is too small to draw general conclusions.

### 4.3 Excitonic Energy Splitting

Our analysis in Section 3.2 and 3.3 suggests that the perturbation expression for the excitonic coupling is not fully separable and that its computation from transition densities of the isolated systems will lead to deviations unless for the full coupled-cluster expansion. Here, we compare the excitonic energy splitting for dimers of Ne, CO and C_2_H_4_, as described in Section 4.1.

Figure 1 shows the energy splitting at the CCSD level, either computed by solving for the two lowest excited states of the supersystem, or by coupling the transition densities of the monomers. The latter either employs the full transition density coupled via the Coulomb (and exchange) integrals, or the dipole approximation. In the ideal case of a fully separable theory, all three models are expected to coincide for large separation *R*. Figure 1 employs a doubly logarithmic scale, which maps the *R* ^–3^ behavior of the dipolar coupling to a line with slope −3. Indeed, all three methods for computing the excitonic splitting approach this line. For Ne this is the case for nearly the entire considered range from 8 to 200 Å, while the dipolar or quadrupolar characters of CO and C_2_H_4_ induce slightly larger deviations, in particular at short distances. In case of C_2_H_4_, the π systems of the molecules start to overlap at distances <15 Å due to the very diffuse character of the excited state. As a consequence, the exchange terms become very strong, but lead to artifacts when used without accounting for overlap and charge-transfer contributions. In fact, in this case the neglect of the exchange terms leads to better results at short distances, see Figure 1c, but this issue is not the focus of the present study.

**Figure 1:**
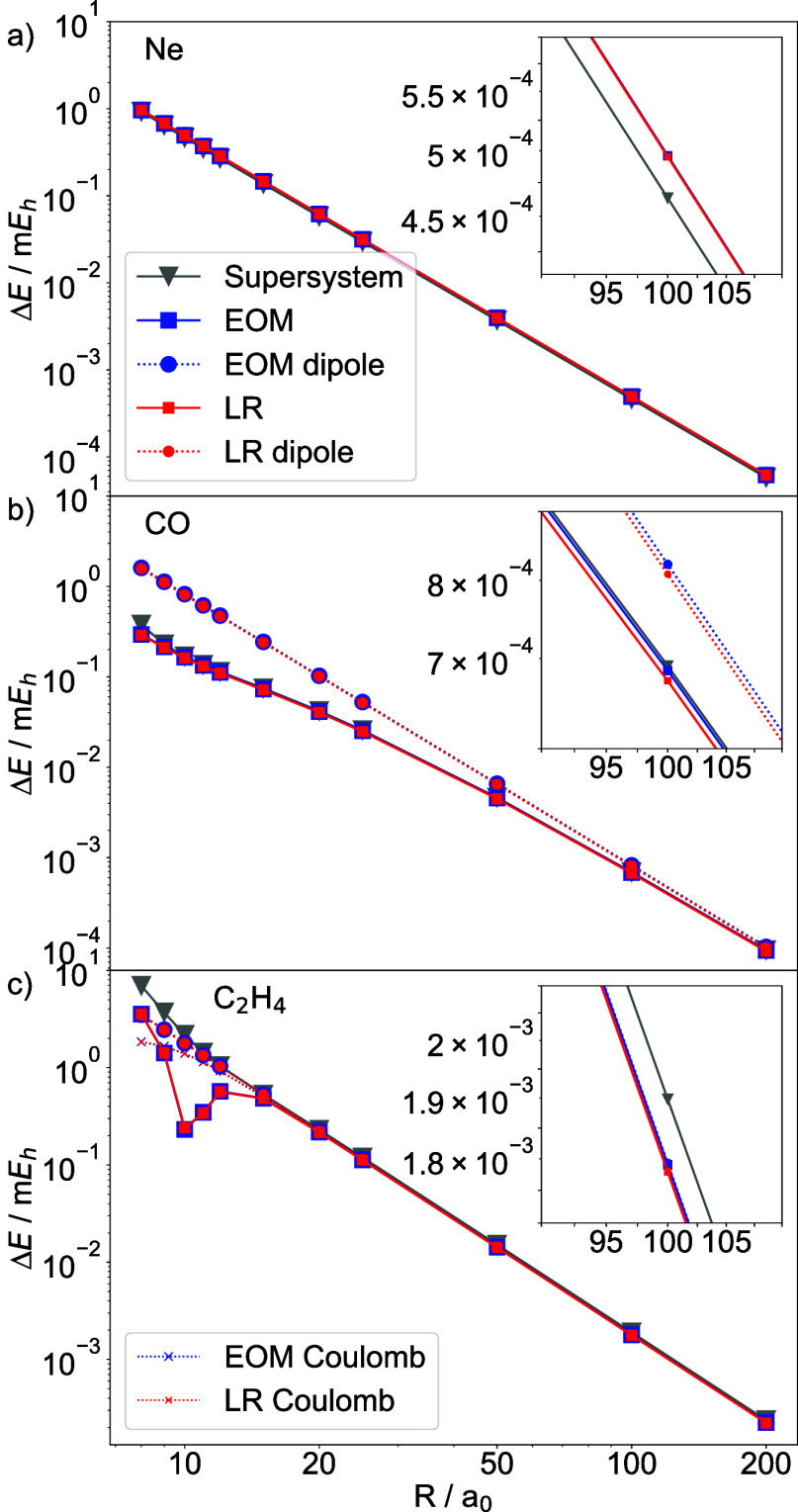
Run of the energy splitting computed at the CCSD level for (a) neon, (b) CO, and (c) ethylene dimers. The computations either use the supersystem approach or the coupling of subsystems. In the latter case, the transition densities from either EOM-CC computations (label ‘EOM’) or CC-LR computations (label ‘LR’) are coupled. For comparison, also the asymptotic dipole couplings based on the transition dipoles from either EOM-CC or CC-LR are given. In panel c), also the results omitting the exchange contribution (label ‘Coulomb’) are shown, as the exchange contributions seem to lead to artifacts.

More importantly, we find for large distances a tiny but notable offset between the predicted energy splittings from the supersystem and the fragment approaches (see insets of Figure 1). For Ne and C_2_H_4_, the dipole and full Coulomb coupling results coincide at this distance, but there is a clearly visible deviation from the supersystem result and, better visible for C_2_H_4_, there is also a slight difference between the EOM and LR results. For CO, interestingly, the Coulomb coupling expression using the EOM transition density matches quite well with the supersystem result, while the LR transition density deviates a little more. In this case, the dipole approximation slightly overestimates the splitting, even at 100 *a*_0_ separation, therefore we will mainly focus our analysis on the full Coulomb coupling of the transition densities.

Despite all the variations in all the presented results, this set of computations at the CCSD level already clearly illustrates the problem expected from our formal analysis: The fragment approaches do not converge to the supersystem result, even for large separations of the monomers, due to the incomplete separability at the truncated coupled-cluster level.

As the next step, we compare the outcomes at different levels of the coupled-cluster hierarchy. To this end, we define a squared effective transition dipole moment (dipole transition strength) by requiring

$$\left|\kappa |\frac{|\mu_{01}^{\mathrm{eff}}{|}^{2}}{R^{3}}=|V_{\mathrm{AB}}\right|$$ 55

at a selected distance *R*, where *V*_AB_ is either computed as half of the energy splitting of the supersystem, or by coupling transition densities via the Coulomb operator. The orientation factor κ is +1 for the cases, in which the transition dipole moment is perpendicular to the intermolecular axis (Ne and CO), and –2 for those cases, in which it is parallel to the axis (C_2_H_4_), compare also eq 22. We choose *R* = 20 Å, as for all cases the supersystem and the Coulomb coupling approach show nearly the same slope at this distance; therefore we can expect that there are only negligible higher-order contributions to intermolecular perturbation theory, see Figure 1.

The resulting effective dipole transition strengths are compared in Figure 2. We find a clear convergence pattern along the coupled-cluster hierarchy, and deviations between the different approaches diminish as the cluster expansion becomes more complete. For the Ne-dimer, a full CCSDTQ computation for the supersystem was feasible and demonstrates nearly perfect agreement with the fragment approaches. Interestingly, for (CO)_2_, the deviations between the supersystem approach and the coupled transition densities remain small all along the coupled-cluster hierarchy.

**Figure 2:**
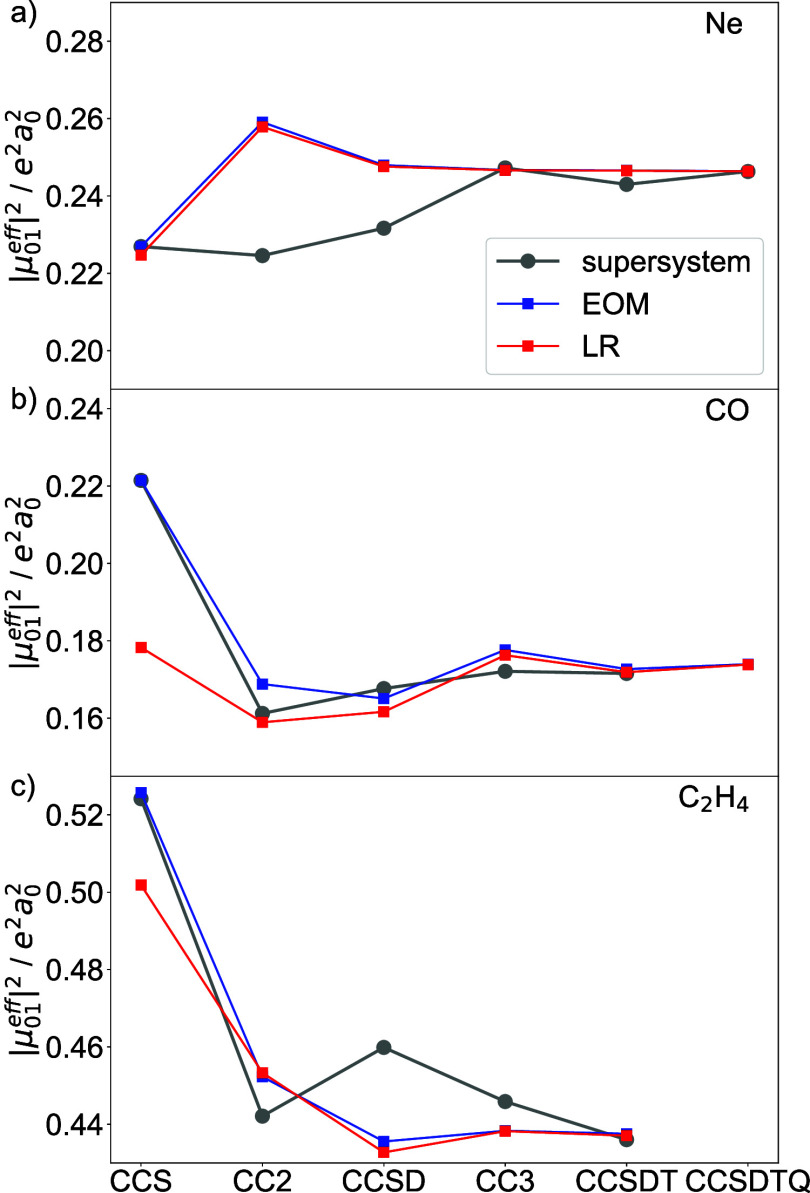
Convergence of effective coupling strength, eq 55, along the coupled-cluster hierarchy. We report the effective dipole coupling strength at a separation of 20 *a*_0_ (see text). The coupling is either obtained from the splitting in the supersystem computation or from the coupling of the transition densities computed either in the EOM or LR formalism. (a) Neon dimer, (b) CO dimer, and (c) C_2_H_4_ dimer.

The CCS approach is again a special case. As discussed before, it equals CIS in the EOM formulation. This is an effective one-electron theory without dynamic correlation and it separates exactly. This is mainly due to the simple structure of the theory, in which the CI vector and the one-particle transition density are identical. The additional LR contribution of CCS has an adverse effect here, in the sense that it destroys separability. On the other hand, for CO and C_2_H_4_, the CCS excitonic splittings are a bit closer to the limit (compare a similar observation for the CCS oscillator strength), but clearly the set of examples is too small to draw further conclusions.

The deviations between the supersystem and the transition-density coupling approaches are quite the same for the widely used CC2 method and the CCSD method, and are on the same order of magnitude as the deviations from the putative full CC limit. With some caution, due to the limited amount of examples, it may be stated that the fragment based computations appear to converge more smoothly to the full CC limit. Concerning the two approaches, EOM and LR theory, there is no clear evidence for a better performance of either of them.

Figure 3 shows a further set of results for Neon dimer using the CCSD method and larger basis sets up to the aug-cc-pVQZ level. The curves run nearly parallel to each other and the relative error remains at the value observed for the small basis sets in the previous paragraph. The tests clearly demonstrate that the basis set incompleteness has no significant impact on the deviations between the supersystem and the transition-density coupling approaches. This is already expected from the theoretical analysis and we therefore confine this demonstration to the case of Neon dimer.

**Figure 3:**
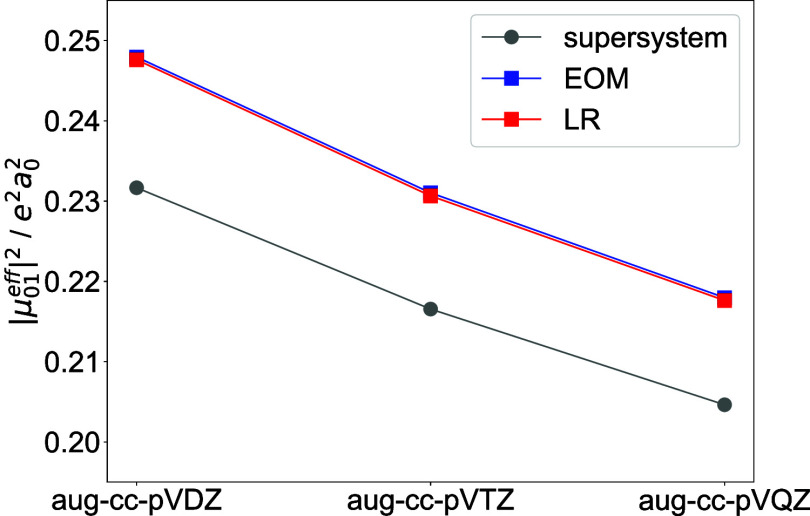
Basis set convergence of the CCSD effective coupling strength, eq 55, for the case of Neon dimer at a separation of 20 *a*_0_. The coupling is either obtained from the splitting in the supersystem computation or from the coupling of the transition densities computed either in the EOM or LR formalism.

Turning back to the comparison of the different methods of the coupled-cluster hierarchy, a slightly different view of the results is given in Figure 4. Here, we analyze the relative error in the excitonic splitting predicted from the coupling of the subsystems, relative to result of the supersystem computation, for all considered separations of the subsystems.

**Figure 4:**
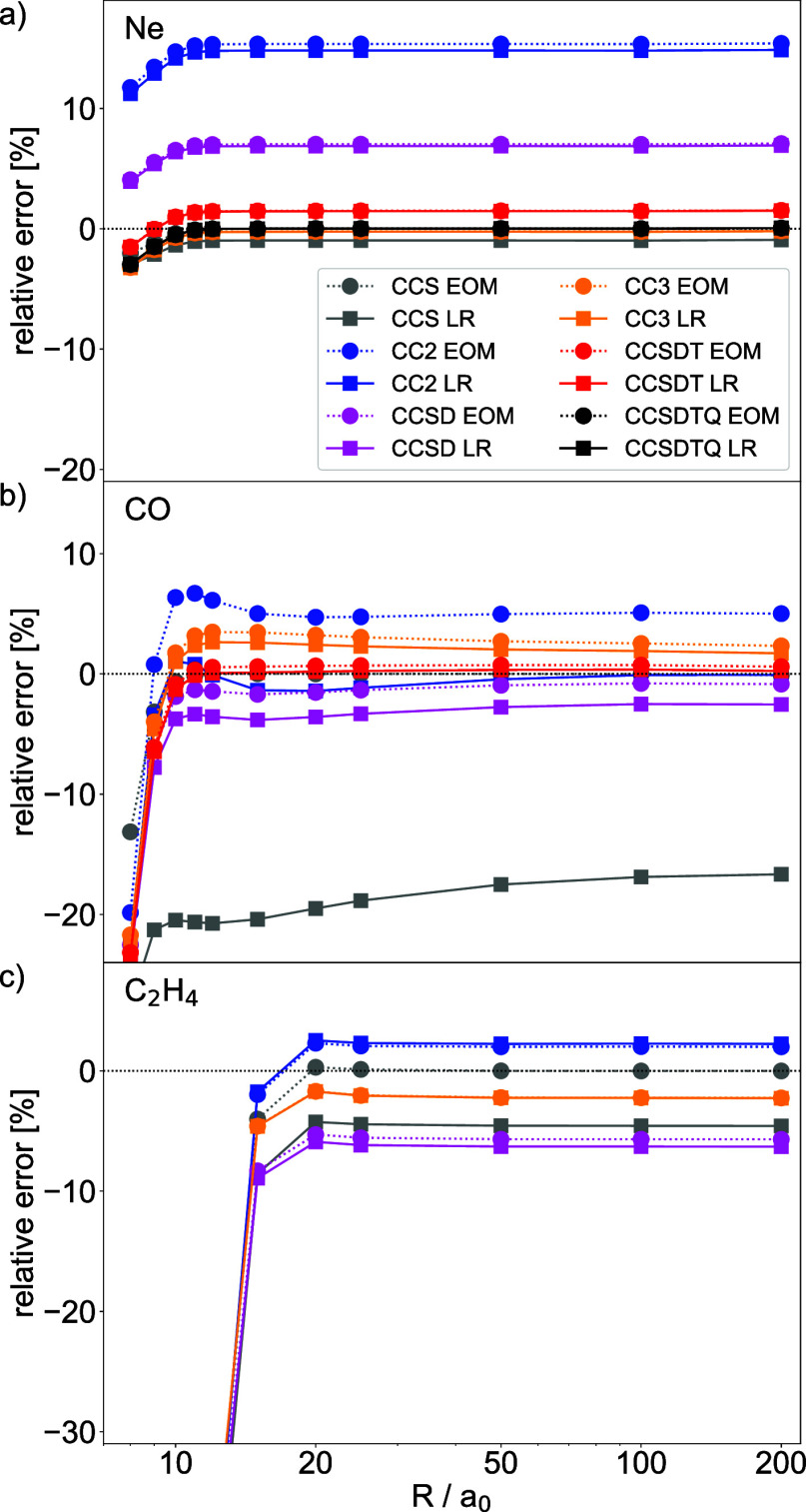
Relative deviation of the energy splitting from coupled transition densities *V*_AB_^c^ to the supersystem computation at the same level of theory, *V*_A_^s^, defined as Δ = (*V*_AB_^c^ – *V*_AB_^s^)/*V*_AB_^s^ ∗ 100*%*. (a) Neon dimer, (b) CO dimer, and (c) C_2_H_4_ dimer.

While the error at short separations is dominated by missing additional terms, it becomes nearly constant beyond 20 Å, indicating that the previous analysis in Figure 2 will be very similar for all longer distances. From this graph, we can quantify the size of the separability error for the different coupled-cluster approaches. It is largest for CCS in the LR formalism, particularly for CO (while CIS is exact, as noted above). For CC2 we find a separability error of 5–15%, for CCSD it ranges around 5%, while for CC3 it is well below 5%. As stated already before, no evidence is found that either the EOM or LR formalism lead to smaller separability errors.

### 4.4 Larger Systems

In order to quantify the impact of our findings in applications to large systems, we carried out further computations at the CC2 level of theory. To this end, the efficient density-fitting based implementation of CC2 in the Turbomole package was employed.^52,53^ As Turbomole only implements the LR formalism, we will only focus on this approach in the present section. Unfortunately, the approach for computing the Coulomb coupling of transition densities (as used in ref 30) is not supported in the versions of Turbomole available to the present author, therefore we have to estimate the error from the dipolar coupling limit for very large intermolecular distances. We again use symmetric dimers of the considered molecules, which allows to extract from the energy splitting a reference value for the excitonic coupling.

As a first test, we consider a coplanar dimer of Butadiene. Using the aug-cc-pVDZ basis set, we can also carry out computations with the setup from the previous section, which allows to assess any impact of the density fitting and the dipole approximation. Unsurprisingly, the differences due to the density fitting approximation are very small for both the excitation energies and the transition dipole moments, as shown in the Supporting Information and in line with previous studies.^52,53^ The computations also demonstrate that the differences between the supersystem computation and the Coulomb-interaction of transition densities become more or less constant from 25 *a*_0_ on, while the dipolar computations converge a bit more slowly. Nevertheless, both coupling approaches indicate a deviation of of about 1.7% from the supersystem splitting in the long-range limit.

For the further computations, we changed to the def2-TVZPP basis set, which is a typical choice for studying valence excited states of larger molecules using the CC2 method. The def2-TVZPP basis set lacks the diffuse functions of aug-cc-pVDZ, but allows for a more flexible description of the valence space. As shown in Table 3, the deviations between the electronic excitonic spliting from the supersystem computations and that from the dipolar expression are a bit smaller than observed for the aug-cc-pVDZ basis set (a bit less than one percent in the long-range limit), but there remains a nonzero offset. The deviations are larger at shorter distances, which is mainly due to the dipolar approximation, as discussed above.

**Table 3:** Excitonic Splitting of Three Larger Chromophore Dimers Computed at the DF-CC2 Level of Theory^a^

system	*R*/*a*_0_	*R*/nm	Δ*E*/cm^–1^	Δ*E*_dip_/cm^–1^	rel. err./%
butadiene	25	1.32	137.19	143.23	4.4
	50	2.65	17.62	17.90	1.6
	100	5.29	2.22	2.24	1.0
	200	10.58	0.28	0.28	0.8
cumarine 120	25	1.32	76.03	107.62	41.6
	50	2.65	10.73	13.45	25.4
	100	5.29	1.39	1.68	21.3
	200	10.58	0.17	0.21	20.3
PDI	25	1.32	183.83	320.13	74.1
	50	2.65	30.07	40.02	33.1
	100	5.29	4.08	5.00	22.5
	200	10.58	0.52	0.63	19.8

a The value of a dimer computation Δ*E* is compared to the value computed from the transition dipole moments Δ*E*_dip_ using the LR formalism.

As two larger examples, we chose two dye molecules, Cumarin 120 (7-amino-4-methyl-cumarine), and PDI (perylenediimide), again considering a coplanar arrangement for simplicity. For rylene dyes, an earlier study^30^ had already established that the dipolar approximation significantly overshoots the coupling for coplanar arrangements and that the dipolar limit is only reached for large distances >10 nm. This is confirmed by the values in Table 3 and also applies to the cumarine dye, although to a lesser extent.

In Figure 5, the relative deviations of the dipolar and supersystem splittings are plotted as a function of the intermolecular separation. The graph clearly shows that the deviation will not converge to zero for large distances and we use the deviation at 200 *a*_0_ (10.6 nm) as a proxy for the error expected for the full Coulomb coupling. Note that the latter is expected to work well for much shorter distances such as 25 *a*_0_ (1.3 nm), as demonstrated by previous examples on smaller molecules.

**Figure 5:**
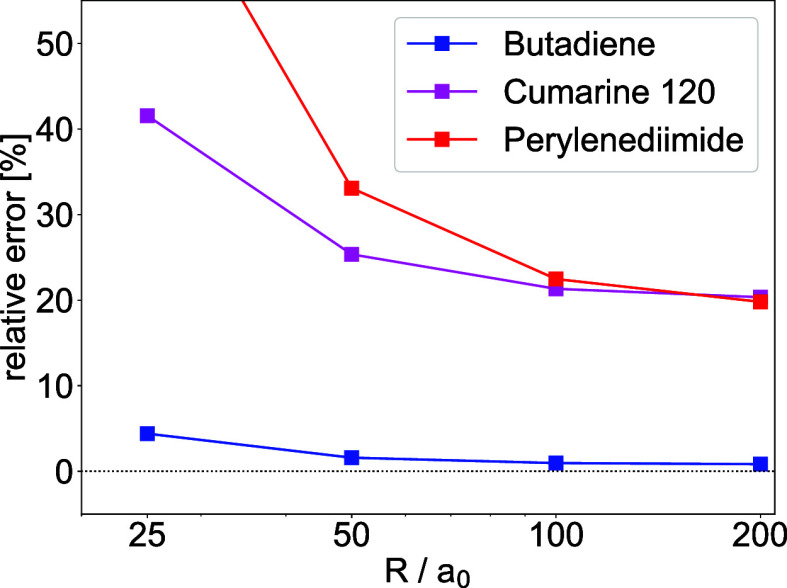
Relative deviation of the energy splitting from coupled transition dipole moments to the supersystem computation at the DF-CC2/def2-TZVPP level of theory for three larger systems.

The results for Cumarine 120 and PDI therefore indicate a significant nonseparability error of approximately 20% for CC2, which is larger that observed in our previous examples. We can estimate that the excitonic splitting computed from the transition densities (and full Coulomb coupling) might overestimate the actual splitting (184 cm^–1^) by 30 to 40 cm^–1^. This deviation is certainly disturbing albeit possibly smaller than the expected accuracy of the CC2 method. It will usually be accurate enough for the typical purposes of such a computation, but it should be kept in mind for internal consistency checks, in particular when constructing and benchmarking local approximations to coupled-cluster theory.

We conclude this section with reporting some observations made for the ADC(2) method,^61,62^ that is very closely related to CC2 and is often used as a slightly more cost-effective alternative. We made some exploratory computations for the PDI dimer (see Supporting Information for details), which indicate larger deviations than those seen for CC2. However, it has to be taken into account that the Turbomole implementation of ADC(2),^63^ for reasons of computational efficiency, does not evaluate the full transition moment expression devised for ADC(2),^61,62^ but omits all parts that require the second-order perturbed ground state wave function. Therefore, (and also as we have not carried out the full theoretical analysis) we cannot give a full account of the separability of the ADC(2) two-particle transition density.

## 5 Conclusions

The separability of the equation-of-motion (EOM) and linear-response (LR) formulations of coupled-cluster (CC) theory for excited states was analyzed, with a focus on excitonic coupling matrix elements. It was shown that the two-electron transition density that formally describes the coupling between two excitations localized on different molecules does not exactly separate into a product of one-electron transition densities. The main source of the problem lies in the linear nature of the projection manifold, which results in disconnected contributions to the left-hand eigenvectors and Lagrange multiplier vectors. These contributions do formally factorize into contributions localized on either fragment, but will only be equivalent to the product space of the fragment basis in the limit of a full coupled-cluster expansion. The analysis leads to similar conclusions both for the EOM and LR formulation of the theory, with no clear formal advantage of either approach. The provided numerical examples show that deviations indeed exist between supermolecular and fragment-based computations of excitonic coupling matrix elements. The deviations are on the order of 10% for low levels of approximation like CC2 and CCSD and are strongly reduced in CCSDT or CCSDTQ reference computations. Exploratory computations for large chromophores such as perylene or cumarine dyes were performed at the CC2 level and show that the dipolar coupling computed from transition moments of the individual fragments can deviate by up to 20% from the dipolar limit extrapolated from supermolecular computations. The problem likely transfers to related excited state methods like ADC­(2). Effective one-particle methods like time-dependent density functional theory will not suffer from this problem, in analogy to our observations for the configuration interaction singles (CIS) method, which has been evaluated in this work as a lowest-level member of the EOM-CC family. As a main conclusion, one has to be clear that fragment based approaches to coupled-cluster computations of molecular aggregates will not agree exactly with supermolecular computations, even in the large-separation limit, which can be of some relevance for designing and benchmarking local correlation methods. For practical computations, however, the deviations appear less critical, as they will likely not deteriorate the overall accuracy of the method.

## Supplementary Material





## Data Availability

The data that supports the findings of this study are available within the article and its Supporting Information material.

## Notes

Additional notes on the block diagonal form of the supersystem EOM matrix, geometries and sample inputs for all computations, additional tests for butadiene regarding dipolar couplings and the density fitting approximation, comparison of CC2 and ADC(2) results (PDF)
Results of all calculations (XLSX)
