# Supplementary material for: Separability of the Coupled-Cluster Excited State Equations: The Case of Excitonic Couplings
Source: J Phys Chem A. 2026 Jun 4;130(24):4657–69. doi: 10.1021/acs.jpca.6c00608 (PMC13288680; doi:10.1021/acs.jpca.6c00608)
Supplement: Supplementary file 1 [file jp6c00608_si_001.pdf]

Supporting Information for: Separability of the Coupled-Cluster Excited State Equations: The Case of Excitonic Couplings

Andreas Köhn*

Institute for Theoretical Chemistry, University of Stuttgart, Paffenwaldring 55, D-70569 Stuttgart, Germany

E-mail: koehn@theochem.uni-stuttgart.de

Contents

S1 Theory Notes **S3**
- S1.1 Disconnected terms in EOM transition moment S3
- S1.2 Non-zero matrix elements in EOM supersystem matrix S3
- S1.3 Eigenvectors of EOM supersystem matrix S4

S2 Geometries **S6**
- S2.1 Ne S6
- S2.2 CO S6
- S2.3 C_2H_4 . . . . S7
- S2.4 trans-Butadiene S7
- S2.5 Cumarine 120 . . . . S8
- S2.6 Perylenediimide S9

S3 Sample Inputs **S10**
- S3.1 C_2H_4: Monomer input S10
- S3.2 C_2H_4: Dimer S11
- S3.3 Settings in Turbomole calculations S14

S4 Comparison of approaches for trans-butadiene **S15**
S4.1 Test of dipolar approximation S15
S4.2 Test of Density Fitting approximation S16

S5 Comparison of CC2 and ADC(2) **S16**

S6 Raw Data **S18**

S1 Theory Notes

The discussion uses the notation introduced in the main text. The argument follows closely the considerations of Stanton, *J. Chem. Phys.* **1994**, *101*, 8928–8937.

S1.1 Disconnected terms in EOM transition moment

The EOM $0n$ transition moment is

$$X_{0n}^{\mathrm{EOM}} = \sum_{\sigma} \langle \Phi_0 | (1+\hat{\Lambda}) [\bar{X}, \hat{\tau}_\sigma] | \Phi_0 \rangle R_\sigma^{(n)}, \tag{S1}$$

where the summation over σ includes all configurations including the reference determinant, while the LR $0n$ transition moment reads:

$$X_{0n}^{\mathrm{LR}} = \sum_{\sigma>0} \langle \Phi_0 | (1+\hat{\Lambda}) [\bar{X}, \hat{\tau}_\sigma] | \Phi_0 \rangle R_\sigma^{(n)} + \sum_{\rho>0} M_\rho^{(0n)} \langle \Phi_\rho | \bar{X} | \Phi_0 \rangle, \tag{S2}$$

where the summation over σ only includes excited configurations. In order to better expose the extra contributions of the EOM expression that are not contained in the first part of the LR expression, the difference of these terms can be rewritten in the following way (using the resolution of the identity $1 = \sum_{\rho>0} |\Phi_\rho\rangle\langle\Phi_\rho| + |\Phi_0\rangle\langle\Phi_0|$ in the second equality, see also Koch *et al. J. Chem. Phys.* **1994**, *100*, 4393–4400):

$$\sum_{\sigma} \langle \Phi_0 | (1+\hat{\Lambda}) \bar{X} \hat{\tau}_\sigma | \Phi_0 \rangle R_\sigma^{(n)} - \sum_{\sigma>0} \langle \Phi_0 | (1+\hat{\Lambda}) [\bar{X}, \hat{\tau}_\sigma] | \Phi_0 \rangle R_\sigma^{(n)} \tag{S3}$$

$$= \sum_{\sigma>0} \langle \Phi_0 | (1+\hat{\Lambda}) \hat{\tau}_\sigma \bar{X} | \Phi_0 \rangle R_\sigma^{(n)} + \langle \Phi_0 | (1+\hat{\Lambda}) \bar{X} | \Phi_0 \rangle R_0^{(n)} \tag{S4}$$

$$= \sum_{\rho,\sigma>0} R_\sigma \langle \Phi_0 | \hat{\Lambda} \hat{\tau}_\sigma | \Phi_\rho \rangle \langle \Phi_\rho | \bar{X} | \Phi_0 \rangle + \sum_{\sigma>0} R_\sigma \langle \Phi_0 | \hat{\Lambda} \hat{\tau}_\sigma | \Phi_0 \rangle \langle \Phi_0 | \bar{X} | \Phi_0 \rangle + \langle \Phi_0 | (1+\hat{\Lambda}) \bar{X} | \Phi_0 \rangle R_0^{(n)}\,. \tag{S5}$$

It is obvious that these terms are partially disconnected. In particular, they contain the contribution $\langle \Phi_\rho | \bar{X} | \Phi_0 \rangle$ which is the coupled-cluster residual evaluated for the perturbation and represents, in a way, a response of the cluster amplitudes towards the perturbing operator. In the LR expression, a similar contribution comes from the last term of eq. S2 (see also main text).

S1.2 Non-zero matrix elements in EOM supersystem matrix

The derivation exploits that the similarity transformed supersystem Hamiltonian is separable

$$\bar{H} = \bar{H}_{\mathrm{A}} + \bar{H}_{\mathrm{B}} \tag{S6}$$

and therefore does not support matrix elements between configurations with different electron distributions between subsystem A and B. In addition the orthogonality of configurations is

used. For clarity, we express the configurations as direct products of local configurations:

$$|\Phi_0\rangle = |\Phi_0^{\mathrm{A}}\Phi_0^{\mathrm{B}}\rangle, \tag{S7}$$
$$|\Phi_{\sigma_{\mathrm{A}}}\rangle = |\Phi_{\sigma_{\mathrm{A}}}^{\mathrm{A}}\Phi_0^{\mathrm{B}}\rangle, \tag{S8}$$
$$|\Phi_{\sigma_{\mathrm{B}}}\rangle = |\Phi_0^{\mathrm{A}}\Phi_{\sigma_{\mathrm{B}}}^{\mathrm{B}}\rangle, \tag{S9}$$
$$|\Phi_{\sigma_{\mathrm{X}}}\rangle = |\Phi_{\sigma_{\mathrm{X,A}}}^{\mathrm{A}}\Phi_{\sigma_{\mathrm{X,B}}}^{\mathrm{B}}\rangle. \tag{S10}$$

The non-zero blocks of the EOM matrix are therefore given by:

$$\bar{H}_{00}: \ \langle\Phi_0|\bar{H}_{\mathrm{A}}+\bar{H}_{\mathrm{B}}|\Phi_0\rangle = E_0^{\mathrm{A}}+E_0^{\mathrm{B}}, \tag{S11}$$
$$\bar{\mathbf{H}}_{0\mathrm{A}}: \ \langle\Phi_0|\bar{H}_{\mathrm{A}}+\bar{H}_{\mathrm{B}}|\Phi_{\sigma_{\mathrm{A}}}\rangle = \langle\Phi_0|\bar{H}_{\mathrm{A}}|\Phi_{\sigma_{\mathrm{A}}}\rangle, \tag{S12}$$
$$\bar{\mathbf{H}}_{0\mathrm{B}}: \ \langle\Phi_0|\bar{H}_{\mathrm{A}}+\bar{H}_{\mathrm{B}}|\Phi_{\sigma_{\mathrm{B}}}\rangle = \langle\Phi_0|\bar{H}_{\mathrm{B}}|\Phi_{\sigma_{\mathrm{B}}}\rangle, \tag{S13}$$
$$\bar{\mathbf{H}}_{\mathrm{AA}}: \ \langle\Phi_{\rho_{\mathrm{A}}}|\bar{H}_{\mathrm{A}}+\bar{H}_{\mathrm{B}}|\Phi_{\sigma_{\mathrm{A}}}\rangle = \langle\Phi_{\rho_{\mathrm{A}}}|\bar{H}_{\mathrm{A}}|\Phi_{\sigma_{\mathrm{A}}}\rangle + E_0^{\mathrm{B}}\delta_{\rho_{\mathrm{A}}\sigma_{\mathrm{A}}}, \tag{S14}$$
$$\bar{\mathbf{H}}_{\mathrm{BB}}: \ \langle\Phi_{\rho_{\mathrm{B}}}|\bar{H}_{\mathrm{A}}+\bar{H}_{\mathrm{B}}|\Phi_{\sigma_{\mathrm{B}}}\rangle = \langle\Phi_{\rho_{\mathrm{B}}}|\bar{H}_{\mathrm{B}}|\Phi_{\sigma_{\mathrm{B}}}\rangle + E_0^{\mathrm{A}}\delta_{\rho_{\mathrm{B}}\sigma_{\mathrm{B}}}, \tag{S15}$$
$$\bar{\mathbf{H}}_{\mathrm{AX}}: \ \langle\Phi_{\rho_{\mathrm{A}}}|\bar{H}_{\mathrm{A}}+\bar{H}_{\mathrm{B}}|\Phi_{\sigma_{\mathrm{X}}}\rangle = \langle\Phi_{\rho_{\mathrm{A}}}^{\mathrm{A}}\Phi_0^{\mathrm{B}}|\bar{H}_{\mathrm{B}}|\Phi_{\sigma_{\mathrm{X,A}}}^{\mathrm{A}}\Phi_{\sigma_{\mathrm{X,B}}}^{\mathrm{B}}\rangle, \tag{S16}$$
$$\bar{\mathbf{H}}_{\mathrm{BX}}: \ \langle\Phi_{\rho_{\mathrm{B}}}|\bar{H}_{\mathrm{A}}+\bar{H}_{\mathrm{B}}|\Phi_{\sigma_{\mathrm{X}}}\rangle = \langle\Phi_0^{\mathrm{A}}\Phi_{\rho_{\mathrm{B}}}^{\mathrm{B}}|\bar{H}_{\mathrm{A}}|\Phi_{\sigma_{\mathrm{X,A}}}^{\mathrm{A}}\Phi_{\sigma_{\mathrm{X,B}}}^{\mathrm{B}}\rangle, \tag{S17}$$
$$\bar{\mathbf{H}}_{\mathrm{XX}}: \ \langle\Phi_{\rho_{\mathrm{X}}}|\bar{H}_{\mathrm{A}}+\bar{H}_{\mathrm{B}}|\Phi_{\sigma_{\mathrm{X}}}\rangle = \langle\Phi_{\rho_{\mathrm{X,A}}}^{\mathrm{A}}\Phi_{\rho_{\mathrm{X,B}}}^{\mathrm{B}}|\bar{H}_{\mathrm{A}}+\bar{H}_{\mathrm{B}}|\Phi_{\sigma_{\mathrm{X,A}}}^{\mathrm{A}}\Phi_{\sigma_{\mathrm{X,B}}}^{\mathrm{B}}\rangle. \tag{S18}$$

S1.3 Eigenvectors of EOM supersystem matrix

According to the analysis above, the block-matrix form of the EOM matrix for the supersystem reads

$$\bar{\mathbf{H}} = \begin{pmatrix} \bar{H}_{00} & \bar{\mathbf{H}}_{0\mathrm{A}} & \bar{\mathbf{H}}_{0\mathrm{B}} & \mathbf{0} \\ \mathbf{0} & \bar{\mathbf{H}}_{\mathrm{AA}} & \mathbf{0} & \bar{\mathbf{H}}_{\mathrm{AX}} \\ \mathbf{0} & \mathbf{0} & \bar{\mathbf{H}}_{\mathrm{BB}} & \bar{\mathbf{H}}_{\mathrm{BX}} \\ \mathbf{0} & \mathbf{0} & \mathbf{0} & \bar{\mathbf{H}}_{\mathrm{XX}} \end{pmatrix}. \tag{S19}$$

This leads to the following equations for the right-hand side eigenvalue problem:

$$(\bar{H}_{00}-E_n)R_0 + \bar{\mathbf{H}}_{0\mathrm{A}}\mathbf{R}_{\mathrm{A}} + \bar{\mathbf{H}}_{0\mathrm{B}}\mathbf{R}_{\mathrm{B}} + \bar{\mathbf{H}}_{0\mathrm{X}}\mathbf{R}_{\mathrm{X}} = 0, \tag{S20}$$
$$(\bar{\mathbf{H}}_{\mathrm{AA}}-E_n\mathbf{1}_{\mathrm{A}})\mathbf{R}_{\mathrm{A}} + \bar{\mathbf{H}}_{\mathrm{AX}}\mathbf{R}_{\mathrm{X}} = 0, \tag{S21}$$
$$(\bar{\mathbf{H}}_{\mathrm{BB}}-E_n\mathbf{1}_{\mathrm{B}})\mathbf{R}_{\mathrm{B}} + \bar{\mathbf{H}}_{\mathrm{BX}}\mathbf{R}_{\mathrm{X}} = 0, \tag{S22}$$
$$(\bar{\mathbf{H}}_{\mathrm{XX}}-E_n\mathbf{1}_{\mathrm{X}})\mathbf{R}_{\mathrm{X}} = 0. \tag{S23}$$

If we suppose that $E_n \equiv E_{\mathrm{A}}$ is an eigenvalue of $\bar{\mathbf{H}}_{\mathrm{AA}}$, then the other equations have only the trivial solutions, that is $\mathbf{R}_X = \mathbf{0}$ and $\mathbf{R}_B = \mathbf{0}$, and the solution has the form

$$\mathbf{R}^{(\mathrm{A})} = \begin{pmatrix} R_0^{(\mathrm{A})} \\ \mathbf{R}_{\mathrm{A}}^{(\mathrm{A})} \\ \mathbf{0} \\ \mathbf{0} \end{pmatrix}. \tag{S24}$$

The analogous argument holds if $E_n \equiv E_\mathrm{B}$ is an eigenvalue of $\bar{\mathbf{H}}_\mathrm{BB}$ and we get

$$\mathbf{R}^{(\mathrm{B})} = \begin{pmatrix} R_0^{(\mathrm{B})} \\ \mathbf{0} \\ \mathbf{R}_\mathrm{B}^{(\mathrm{B})} \\ \mathbf{0} \end{pmatrix} . \tag{S25}$$

The degenerate case, when $\bar{\mathbf{H}}_\mathrm{AA}$ and $\bar{\mathbf{H}}_\mathrm{BB}$ have the same eigenvalue, is unproblematic as there is no direct coupling between these blocks. Therefore, we can always choose the perfectly localised solution.

For the left-hand side eigenvalue problem we obtain these equations: First of all, the scalar component becomes

$$L_0(\bar{H}_{00} - E_n) = 0. \tag{S26}$$

This equation has only a nontrivial solution for the ground state when $\bar{H}_{00} = E_0$, for all excited states $L_0 = 0$ and we can directly drop it from the other equations. We then have

$$\mathbf{L}_\mathrm{A}(\bar{\mathbf{H}}_\mathrm{AA} - E_n \mathbf{1}_\mathrm{A}) = 0, \tag{S27}$$

$$\mathbf{L}_\mathrm{B}(\bar{\mathbf{H}}_\mathrm{BB} - E_n \mathbf{1}_\mathrm{B}) = 0, \tag{S28}$$

$$\mathbf{L}_\mathrm{A}\bar{\mathbf{H}}_\mathrm{BX} + \mathbf{L}_\mathrm{B}\bar{\mathbf{H}}_\mathrm{BB} + \mathbf{L}_\mathrm{X}(\bar{\mathbf{H}}_\mathrm{XX} - E_n \mathbf{1}_\mathrm{X}) = 0. \tag{S29}$$

If we assume that $E_n \equiv E_\mathrm{A}$ is an eigenvalue of $\bar{\mathbf{H}}_\mathrm{AA}$, then the second line has only the trivial solution $\mathbf{L}_\mathrm{B} = \mathbf{0}$ and we are left with the linear set of equations (as discussed in the main text):

$$\mathbf{L}_\mathrm{X}^{(\mathrm{A})}(\bar{\mathbf{H}}_\mathrm{XX} - E_\mathrm{A} \mathbf{1}_\mathrm{X}) = -\mathbf{L}_\mathrm{A}^{(\mathrm{A})}\bar{\mathbf{H}}_\mathrm{AX} . \tag{S30}$$

The analogous discussion applies for the eigenvectors of $\bar{\mathbf{H}}_\mathrm{B}$ and again a degeneracy of the two systems does not cause problems. The localised solutions have the eigenvectors:

$$\mathbf{L}^{(\mathrm{A})} = (0, \mathbf{L}_\mathrm{A}^{(\mathrm{A})}, \mathbf{0}, \mathbf{L}_\mathrm{X}^{(\mathrm{A})}), \tag{S31}$$

$$\mathbf{L}^{(\mathrm{B})} = (0, \mathbf{0}, \mathbf{L}_\mathrm{B}^{(\mathrm{B})}, \mathbf{L}_\mathrm{X}^{(\mathrm{B})}) . \tag{S32}$$

If we assume that the X basis is spanned by the complete product basis A$\otimes$B, eq. S30 can be factorised: We suppose that the required solution is $\mathbf{L}_\mathrm{X}^{(\mathrm{A})} = \mathbf{L}_\mathrm{A}^{(\mathrm{A})}\boldsymbol{\Lambda}_\mathrm{B}$ with $E_\mathrm{A} = E_0^\mathrm{A} + E_0^\mathrm{B} + \omega_\mathrm{A}$. As indicated in the main text, the Hamiltonian separates as

$$\bar{\mathbf{H}}_\mathrm{XX} = \tilde{\mathbf{H}}_\mathrm{A} \otimes \mathbf{1}_\mathrm{B} + \mathbf{1}_\mathrm{A} \otimes \tilde{\mathbf{H}}_\mathrm{B}, \tag{S33}$$

with $\tilde{\mathbf{H}}_\mathrm{A} = \bar{\mathbf{H}}_\mathrm{A} - E_0^\mathrm{B}$ and $\tilde{\mathbf{H}}_\mathrm{B} = \bar{\mathbf{H}}_\mathrm{B} - E_0^\mathrm{A}$, and the right-hand can be written as

$$-\mathbf{L}_\mathrm{A}^{(\mathrm{A})}\bar{\mathbf{H}}_\mathrm{AX} = -\mathbf{L}_\mathrm{A}^{(\mathrm{A})} \otimes \bar{\mathbf{H}}_\mathrm{0B}. \tag{S34}$$

Therefore eq. S30 can be rewritten as

$$\mathbf{L}_{\mathrm{A}}^{(\mathrm{A})}\tilde{\mathbf{H}}_{\mathrm{AA}} \otimes \mathbf{\Lambda}_{\mathrm{B}} + \mathbf{L}_{\mathrm{A}}^{(\mathrm{A})} \otimes \mathbf{\Lambda}_{\mathrm{B}}\tilde{\mathbf{H}}_{\mathrm{BB}} - (E_0^{\mathrm{A}} + E_0^{\mathrm{B}} + \omega_{\mathrm{A}})\mathbf{L}_{\mathrm{A}}^{(\mathrm{A})} \otimes \mathbf{\Lambda}_{\mathrm{B}} = -\mathbf{L}_{\mathrm{A}}^{(\mathrm{A})} \otimes \bar{\mathbf{H}}_{0\mathrm{B}} \tag{S35}$$

or

$$\left[\mathbf{L}_{\mathrm{A}}^{(\mathrm{A})}\tilde{\mathbf{H}}_{\mathrm{AA}} - \mathbf{L}_{\mathrm{A}}^{(\mathrm{A})}(E_0^{\mathrm{A}} + \omega_{\mathrm{A}})\right] \otimes \mathbf{\Lambda}_{\mathrm{B}} + \mathbf{L}_{\mathrm{A}}^{(\mathrm{A})} \otimes \left[\mathbf{\Lambda}_{\mathrm{B}}(\tilde{\mathbf{H}}_{\mathrm{BB}} - E_0^{\mathrm{B}}) + \bar{\mathbf{H}}_{0\mathrm{B}}\right] = 0, \tag{S36}$$

which shows that under these assumptions the excited state left-hand EOM equation for $\mathbf{L}_{\mathrm{A}}^{(\mathrm{A})}$ and the Λ equations for subsystem B are recovered.

S2 Geometries

Molpro input format is used, unless noted otherwise. See

`https://www.molpro.net/manual/doku.php?id=molecular_geometry`

for details.

S2.1 Ne

Monomer:

```
geometry={ne}
```

Dimer: (all distances in a_0, `R` can be varied)

```
geometry={ne,0, 0., 0. ,-0.5*R;
          ne,0, 0., 0. , 0.5*R}
```

S2.2 CO

Monomer: (all distances in a_0:)

```
geometry={c,0, 0., 0. ,-1.21806338006687164176;
          o,0, 0., 0. , 0.91354753505015373130}
```

Dimer: (all distances in a_0, `R` can be varied)

```
geometry={c,0, 0., 0. ,-0.5*R - 1.21806338006687164176;
          o,0, 0., 0. ,-0.5*R + 0.91354753505015373130;
          c,0, 0., 0. , 0.5*R + 1.21806338006687164176;
          o,0, 0., 0. , 0.5*R - 0.91354753505015373130}
```

S2.3 C_2H_4

Monomer: (all distances in a_0)

```
geometry={c,0, 0.    , 1.2756, 0.;
          c,0, 0.    ,-1.2756, 0.;
          h,0, 1.7582, 2.3487, 0.;
          h,0, 1.7582,-2.3487, 0.;
          h,0,-1.7582, 2.3487, 0.;
          h,0,-1.7582,-2.3487, 0.}
```

Dimer: (all distances in a_0, R can be varied)

```
geometry={c,0, 0.    , 1.2756, -0.5*R;
          c,0, 0.    ,-1.2756, -0.5*R;
          h,0, 1.7582, 2.3487, -0.5*R;
          h,0, 1.7582,-2.3487, -0.5*R;
          h,0,-1.7582, 2.3487, -0.5*R;
          h,0,-1.7582,-2.3487, -0.5*R;
          c,0, 0.    , 1.2756,  0.5*R;
          c,0, 0.    ,-1.2756,  0.5*R;
          h,0, 1.7582, 2.3487,  0.5*R;
          h,0, 1.7582,-2.3487,  0.5*R;
          h,0,-1.7582, 2.3487,  0.5*R;
          h,0,-1.7582,-2.3487,  0.5*R}
```

S2.4 trans-Butadiene

Monomer: (all distances in a_0)

```
geometry={c,0,  3.44859,   -0.50274,  0.;
          c,0,  0.96103,   -0.98070,  0.;
          c,0, -0.96103,    0.98070,  0.;
          c,0, -3.44859,    0.50274,  0.;
          h,0,  4.14719,    1.41841,  0.;
          h,0,  4.82271,   -2.01088,  0.;
          h,0,  0.30373,   -2.92188,  0.;
          h,0, -0.30373,    2.92188,  0.;
          h,0, -4.14719,   -1.41841,  0.;
          h,0, -4.82271,    2.01088,  0.}
```

Dimer: (all distances in a_0, R can be varied)

```
geometry={c,0,  3.44859,   -0.50274,  -0.5*R;
          c,0,  0.96103,   -0.98070,  -0.5*R;
          c,0, -0.96103,    0.98070,  -0.5*R;
          c,0, -3.44859,    0.50274,  -0.5*R;
          h,0,  4.14719,    1.41841,  -0.5*R;
          h,0,  4.82271,   -2.01088,  -0.5*R;
```

```
        h,0,  0.30373,   -2.92188,  -0.5*R;
        h,0, -0.30373,    2.92188,  -0.5*R;
        h,0, -4.14719,   -1.41841,  -0.5*R;
        h,0, -4.82271,    2.01088,  -0.5*R;
        c,0,  3.44859,   -0.50274,   0.5*R;
        c,0,  0.96103,   -0.98070,   0.5*R;
        c,0, -0.96103,    0.98070,   0.5*R;
        c,0, -3.44859,    0.50274,   0.5*R;
        h,0,  4.14719,    1.41841,   0.5*R;
        h,0,  4.82271,   -2.01088,   0.5*R;
        h,0,  0.30373,   -2.92188,   0.5*R;
        h,0, -0.30373,    2.92188,   0.5*R;
        h,0, -4.14719,   -1.41841,   0.5*R;
        h,0, -4.82271,    2.01088,   0.5*R
}
```

S2.5 Cumarine 120

Monomer: (TURBOMOLE format, all distances in a_0)

```
$coord
   -3.41193562611259     -0.56443581615521      0.00000000000000  c
   -4.77182307908844      1.60529397614419      0.00000000000000  c
   -3.61848385221089      4.09394373275415      0.00000000000000  c
   -0.99854675618404      4.16516310891906      0.00000000000000  o
    0.41528435235458      2.00783140114156      0.00000000000000  c
   -0.69365701524379     -0.39615171214549      0.00000000000000  c
    0.93859775253673     -2.48837375341724      0.00000000000000  c
    3.53262958137799     -2.18686067026009      0.00000000000000  c
    4.61492918461885      0.24237695554823      0.00000000000000  c
    3.01724817917853      2.34001091580457      0.00000000000000  c
    7.18948980924448      0.53478711195278      0.00000000000000  n
   -4.73768807604491      6.08417763170423      0.00000000000000  o
   -4.67298103111904     -3.09594385851376      0.00000000000000  c
   -6.81320615734668      1.58882156744443      0.00000000000000  h
    0.15379016331020     -4.37498156914219      0.00000000000000  h
    4.75001457213830     -3.83044604800182      0.00000000000000  h
    3.76331903767255      4.24351192455697      0.00000000000000  h
    7.97228819024132      2.25602794927360      0.00000000000000  h
    8.33343956846865     -0.97016828346051      0.00000000000000  h
   -4.12292337045708     -4.18350653746648     -1.66084294647419  h
   -4.12292337045708     -4.18350653746648      1.66084294647419  h
   -6.71686205687755     -2.88757148921458      0.00000000000000  h
```

Dimer: The same x and y coordinates as above were used for both subsystems and the z coordinates were added $\pm 12.5\ a_0$, $\pm 25\ a_0$, $\pm 50\ a_0$, $\pm 100\ a_0$.

S2.6 Perylenediimide

Monomer: (TURBOMOLE format, all distances in a_0)

```
$coord
   -6.51829388935205     -6.35949564349870      0.00000000000000      h
   -5.44642544080235     -4.59528875487041      0.00000000000000      c
   -9.57188855045433     -2.36836194794594      0.00000000000000      c
   -6.76041363341143     -2.32327854225594      0.00000000000000      c
   -1.38602118855226     -2.35992499560647      0.00000000000000      c
   -5.42060027488874      0.00000000000000      0.00000000000000      c
   -2.79897442539745     -4.60283639433078      0.00000000000000      c
   -2.70929070704180      0.00000000000000      0.00000000000000      c
   -6.76041363341143      2.32327854225594      0.00000000000000      c
   -1.83713188553283     -6.42571790527025      0.00000000000000      h
    1.38602118855226      2.35992499560647      0.00000000000000      c
    1.38602118855226     -2.35992499560647      0.00000000000000      c
   -5.44642544080235      4.59528875487041      0.00000000000000      c
   -9.57188855045433      2.36836194794594      0.00000000000000      c
   -6.51829388935205      6.35949564349870      0.00000000000000      h
   -2.79897442539745      4.60283639433078      0.00000000000000      c
   -1.83713188553283      6.42571790527025      0.00000000000000      h
   -1.38602118855226      2.35992499560647      0.00000000000000      c
    2.79897442539745     -4.60283639433078      0.00000000000000      c
    2.70929070704180      0.00000000000000      0.00000000000000      c
    5.42060027488874      0.00000000000000      0.00000000000000      c
    9.57188855045433     -2.36836194794593      0.00000000000000      c
    5.44642544080235     -4.59528875487041      0.00000000000000      c
    1.83713188553283     -6.42571790527025      0.00000000000000      h
    6.76041363341143     -2.32327854225594      0.00000000000000      c
    6.51829388935205     -6.35949564349870      0.00000000000000      h
    6.76041363341143      2.32327854225594      0.00000000000000      c
    2.79897442539745      4.60283639433078      0.00000000000000      c
    5.44642544080235      4.59528875487041      0.00000000000000      c
    1.83713188553283      6.42571790527025      0.00000000000000      h
    6.51829388935205      6.35949564349870      0.00000000000000      h
    9.57188855045433      2.36836194794593      0.00000000000000      c
   10.82275958842828     -4.30104128321283      0.00000000000000      o
   10.82275958842828      4.30104128321283      0.00000000000000      o
   10.71324989751903      0.00000000000000      0.00000000000000      n
  -10.82275958842828      4.30104128321283      0.00000000000000      o
  -10.82275958842828     -4.30104128321283      0.00000000000000      o
  -10.71324989751903      0.00000000000000      0.00000000000000      n
   12.63903809831449      0.00000000000000      0.00000000000000      h
  -12.63903809831449      0.00000000000000      0.00000000000000      h
```

Dimer: The same x and y coordinates as above were used for both subsystems and the z coordinates were added $\pm 12.5\ a_0$, $\pm 25\ a_0$, $\pm 50\ a_0$, $\pm 100\ a_0$.

S3 Sample Inputs

S3.1 C_2H_4: Monomer input

Molpro:

```
memory,1000,m
gprint,orbitals,civector

basis=aug-cc-pVDZ
nosym

geometry={c,0, 0.    , 1.2756, 0.;
          c,0, 0.    ,-1.2756, 0.;
          h,0, 1.7582, 2.3487, 0.;
          h,0, 1.7582,-2.3487, 0.;
          h,0,-1.7582, 2.3487, 0.;
          h,0,-1.7582,-2.3487, 0.}

{hf}

! generate operators and MO coefficients for export to GeCCo
! (newer Molpro versions need a patch, as these files are not in the work
!  directory by default):
{matrop
load,dmx,oper,dmx
load,dmy,oper,dmy
load,over,s
load,dmz,oper,dmz
load,cmo,orb
write,dmx,AOPROPER,new
write,dmy,AOPROPER
write,dmz,AOPROPER
write,over,AOPROPER
write,cmo,CMOMOL,new}

! run gecco with specified input
{cmrcc,gecco,input='ccsd-ee-tm.inp',sub_scratch=F}
```

GeCCo: Running this input requires at least the version with tag `v2026.1`

```
method
 ! choose excitation level:
 CC maxexc=2
 ! for CC2: CC maxexc=2,truncate=CC2
 ! for CC3: CC maxexc=3,truncate=CC3
 CC NEW
 ! choose EOM or LR:
```

```
 CC xs_formalism=LR
calculate
 excitation sym=(1)
 excitation moments
 properties
 solve non_linear method=diis,maxiter=35,conv=1d-7,maxsub=8
 solve linear maxiter=50,conv=1d-7,maxsub=8
 solve eigen maxiter=50,conv=1d-7,maxsub=8
 routes auto_opt=T
```

This will create in particular the files `export_tm0f` and `export_tmf0`, which contain the transition densities backtransformed to AO basis.

S3.2 C_2H_4: Dimer

Coupling of transition densities (Molpro):

```
memory,1000,m
gprint,orbitals,civector
gdirect

Rarray=[8,9,10,11,12,15,20,25,50,100,200]

! loop over all distances:
do idx=1,#Rarray

R = Rarray(idx)

basis=aug-cc-pVDZ

! define dimer geometry
nosym
noextra
geometry={c,0, 0.    , 1.2756, -0.5*R;
          c,0, 0.    ,-1.2756, -0.5*R;
          h,0, 1.7582, 2.3487, -0.5*R;
          h,0, 1.7582,-2.3487, -0.5*R;
          h,0,-1.7582, 2.3487, -0.5*R;
          h,0,-1.7582,-2.3487, -0.5*R;
          c,0, 0.    , 1.2756,  0.5*R;
          c,0, 0.    ,-1.2756,  0.5*R;
          h,0, 1.7582, 2.3487,  0.5*R;
          h,0, 1.7582,-2.3487,  0.5*R;
          h,0,-1.7582, 2.3487,  0.5*R;
          h,0,-1.7582,-2.3487,  0.5*R}

! ensure (re)computation of (one-electron) integrals
int
```

```
{matrop
! read transition densities (expanded to dimer basis, for fragment A)
read,0fa,file=tm0f_a
read,f0a,file=tmf0_a
! read transition densities (expanded to dimer basis, for fragment B)
read,0fb,file=tm0f_b
read,f0b,file=tmf0_b
! load dipole matrix elements
load,dmx,oper,dmx
load,dmy,oper,dmy
load,dmz,oper,dmz
! compute transition densities for check of densities
trace,t0fax,0fa,dmx
trace,t0fay,0fa,dmy
trace,t0faz,0fa,dmz
trace,tf0ax,f0a,dmx
trace,tf0ay,f0a,dmy
trace,tf0az,f0a,dmz
trace,t0fbx,0fb,dmx
trace,t0fby,0fb,dmy
trace,t0fbz,0fb,dmz
trace,tf0bx,f0b,dmx
trace,tf0by,f0b,dmy
trace,tf0bz,f0b,dmz
! compute coulomb and exchange operators for transition densities on fragment A
coul,j1,0fa
exch,x1,0fa
coul,j2,f0a
exch,x2,f0a
! trace with transition densities on fragment B
trace,ic1,j1,f0b
trace,ix1,x1,f0b
trace,ic2,j2,0fb
trace,ix2,x2,0fb
}

! assemble Coulomb and Coulomb/exchange interaction
cab(idx)=2*ic1
cba(idx)=2*ic2
vab(idx)=2*ic1-ix1
vba(idx)=2*ic2-ix2

enddo

! print a table
table,Rarray,vab,vba,cab,cba
```

Supersystem computation (Molpro): Molecular symmetry is exploited

```
memory,1000,m
gprint,orbitals,civector

basis=aug-cc-pVDZ

bohr
R = 20
geometry={c,0, 0.    , 1.2756, -0.5*R;
          c,0, 0.    ,-1.2756, -0.5*R;
          h,0, 1.7582, 2.3487, -0.5*R;
          h,0, 1.7582,-2.3487, -0.5*R;
          h,0,-1.7582, 2.3487, -0.5*R;
          h,0,-1.7582,-2.3487, -0.5*R;
          c,0, 0.    , 1.2756,  0.5*R;
          c,0, 0.    ,-1.2756,  0.5*R;
          h,0, 1.7582, 2.3487,  0.5*R;
          h,0, 1.7582,-2.3487,  0.5*R;
          h,0,-1.7582, 2.3487,  0.5*R;
          h,0,-1.7582,-2.3487,  0.5*R}

hf

{cmrcc,gecco,input='ccsd-ee.inp',sub_scratch=F}
```

Supersystem computation (GeCCo): Molecular symmetry is exploited

```
method
 CC maxexc=2
 CC NEW
calculate
 excitation sym=(1,0,0,0,1,0,0,0)
 solve non_linear method=diis,maxiter=60,conv=1d-10,maxsub=8
 solve linear maxiter=120,conv=1d-10,maxsub=8
 solve eigen maxiter=120,conv=1d-10,maxsub=8
 routes auto_opt=T
```

S3.3 Settings in Turbomole calculations

The following listing summarises the most important settings from the Turbomole `control` file (here for the perylenediimide monomer):

```
$symmetry d2h
$scfconv   6
$denconv     0.1E-06
$freeze
 implicit core=   30 virt=    0
$ricc2
 cc2
$excitations
 irrep=b3u nexc=1
 spectrum
```

For the dimer computation, more strict convergence thresholds were employed for CC2:

```
$symmetry d2h
$scfconv   6
$denconv     0.1E-06
$freeze
 implicit core=   30 virt=    0
$ricc2
 cc2
 oconv=8
$excitations
 irrep=b3u nexc=1
 irrep=b2g nexc=1
 conv=8
```

S4 Comparison of approaches for trans-butadiene

Trans-butadiene was computed at the CC2/aug-cc-pVDZ level both using the Molpro/GeCCo approach outline above, as well as employing the Turbomole code. In the latter case, the density fitting approximation was employed and only the dipolar approximation was available for the estimation of the excitonic coupling from local transitions.

S4.1 Test of dipolar approximation

In Fig. S1 we plot the relative error $\Delta = (V_{\mathrm{AB}}^{\mathrm{c}} - V_{\mathrm{AB}}^{\mathrm{s}})/V_{\mathrm{AB}}^{\mathrm{s}} \times 100\%$ of the approaches using the coupling of either the transition densities via their full Coulomb potential or their transition dipole moments in comparison to the energy splitting of the supersystem calculation. Both approaches converge to the same result at 100 to 200 a_0, where they deviate by 1.7 % from the supersystem result. Clearly, the Coulomb coupling converges more quickly and gives rather accurate results for shorter distances down to 50 a_0 or even 20 to 25 a_0.

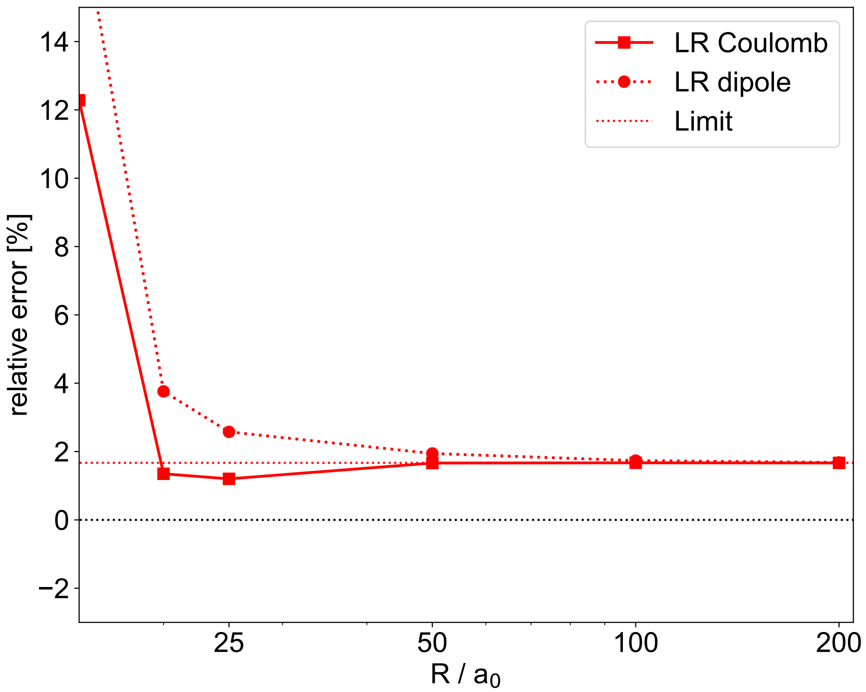

Figure S1: Relative deviation of the energy splitting computed from transition densities in comparison to that of the supersystem computation for butadiene at the CC2/aug-cc-pVDZ level of theory. Either the dipole approximation or the full Coulomb coupling of the transition densities (computed in the linear-response (LR) formalism) was used.

S4.2 Test of Density Fitting approximation

The effect of the density fitting (DF) approximation on the computed transition moments and excitation energy difference is reported in Tabs. S1 and S2, respectively, for the case of butadiene. In case of the transition moments, the deviation of the relevant quantity, the product of $0n$ and $n0$ transition moments, is more than 4 orders of magnitude smaller and will therefore not influence any of our conclusions. Likewise, the DF errors in the excitation energies are unproblematic. For distances beyond 50 a_0, the DF error on the individual transitions is on the order of the energy difference, but the main contribution is a systematic shift of the local transitions. The DF error in the energy splitting is consistently 4 orders of magnitudes smaller than the energy splitting itself.

Table S1: Density fitting error in the transition dipole moments (X and Y direction) of the considered transition of butadiene. The transition moments are computed with CC2/aug-cc-pVDZ in the LR formalism, using either full two-electron integral (GeCCo, based on integrals evaluated in the Molpro code) or using the density fitting approximation (RICC2 module of Turbomole). Note that the transition moments given in RICC2 are based on a different normalisation of the excitation vector. The X_{0n}^{LR} moment of RICC2 has therefore been divided by $\sqrt{2}$, while the X_{n0}^{LR} moment was multiplied by $\sqrt{2}$.

Quantity	GeCCo	RICC2	Diff.
$X_{0n}^{\mathrm{LR}}(X)$ / ea_0	2.05557	2.05547	-1.0×10^{-4}
$X_{0n}^{\mathrm{LR}}(Y)$ / ea_0	−0.20227	−0.20231	-4.1×10^{-5}
$X_{n0}^{\mathrm{LR}}(X)$ / ea_0	2.37279	2.37295	1.7×10^{-4}
$X_{n0}^{\mathrm{LR}}(Y)$ / ea_0	−0.20879	−0.20885	-5.7×10^{-5}
$\sum_{X,Y} X_{0n}^{\mathrm{LR}} X_{n0}^{\mathrm{LR}}$/ $e^2a_0^2$	4.91966	4.91979	1.2×10^{-4}

Table S2: Density fitting error in the excitation energies of the butadiene dimer, computed at the CC2/aug-cc-pVDZ level of theory. The first 3 columns report the computed excitation energies and the resulting splitting computed without DF approximation (GeCCo), while the last 3 columns report the deviations of the DF-based results (RICC2).

R/a_0	ΔE_1 / E_h	ΔE_2 / E_h	ΔE_{21} / E_h	$\Delta_\mathrm{DF}\Delta E_1$ / E_h	$\Delta_\mathrm{DF}\Delta E_2$ / E_h	$\Delta_\mathrm{DF}\Delta E_{21}$ / E_h
25	0.2262322	0.2268461	6.14×10^{-4}	-4.46×10^{-5}	-4.45×10^{-5}	9.65×10^{-8}
50	0.2265006	0.2265778	7.72×10^{-5}	-4.47×10^{-5}	-4.47×10^{-5}	2.63×10^{-9}
100	0.2265344	0.2265440	9.67×10^{-6}	-4.48×10^{-5}	-4.48×10^{-5}	2.86×10^{-10}
200	0.2265386	0.2265398	1.21×10^{-6}	-4.47×10^{-5}	-4.47×10^{-5}	3.99×10^{-11}

S5 Comparison of CC2 and ADC(2)

As the ADC(2) method is often used as a cost-efficient (and Hermitian) alternative to CC2, an exploratory calculation was made for the case of perylenediimide, see Fig. S2. The results

indicate significantly larger deviations for the long range limit, nearly twice as much as for CC2. It should be noted that the TURBOMOLE implementation does not use the full expression for the ADC(2) transition density, but truncates is to those terms that can be computed without knowing the ground state second-order perturbed wavefunction. In this approximation, the transition density expressions are rather similar to those of CC2.

In Tab. S3 we also show the computed transition moments at the CC2 and the ADC(2) level of theory (in the approximation mentioned above). As ADC(2) is a Hermitian theory, the X_{0n} and X_{n0} transition moments are equal.

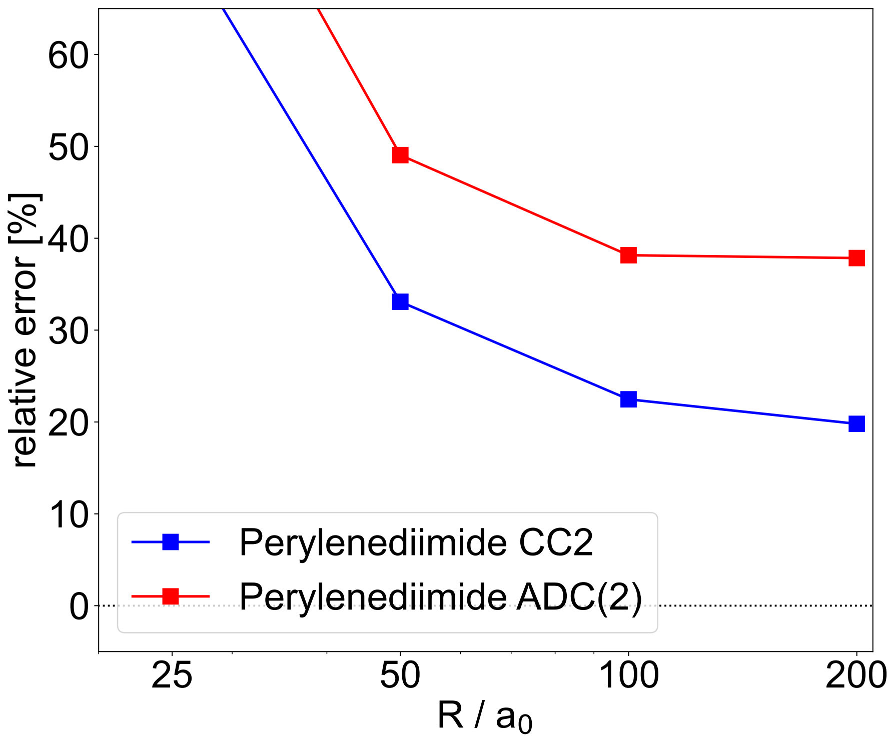

Figure S2: Relative deviation of the energy splitting computed by the coupling of transition dipoles relative to that of the supersystem computation for perylenediimide dimers (co-planar arrangement) at the CC2 and ADC(2) level of theory. The def2-TVZPP basis set was used.

Table S3: Comparison of the computed transition dipole moments (length gauge) and their squares of the lowest singlet transition of perylenediimide. The def2-TVZPP basis set was used.

Method	X_{0n}/ea_0	X_{n0}/ea_0	$X_{0n}X_{n0}/e^2a_0^2$
CC2	-2.965	-3.843	11.396
ADC(2)	-3.497	-3.497	12.228

S6 Raw Data

The raw data is contained in an accompanying spreadsheet file.
